# Targeting ferroptosis for improved radiotherapy outcomes in HPV‐negative head and neck squamous cell carcinoma

**DOI:** 10.1002/1878-0261.13720

**Published:** 2024-09-19

**Authors:** Joo Kyung Noh, Min Kyeong Lee, Yeonseo Lee, Minji Bae, Soonki Min, Moonkyoo Kong, Jung Woo Lee, Su Il Kim, Young Chan Lee, Seong‐Gyu Ko, Seon Rang Woo, Young‐Gyu Eun

**Affiliations:** ^1^ Department of Biomedical Science and Technology, Graduate School Kyung Hee University Seoul Korea; ^2^ Department of Radiation Oncology Kyung Hee University School of Medicine, Kyung Hee University Medical Center Seoul Korea; ^3^ Department of Oral and Maxillofacial Surgery, School of Dentistry Kyung Hee University Seoul Korea; ^4^ Department of Otolaryngology‐Head and Neck Surgery Kyung Hee University School of Medicine, Kyung Hee University Medical Center Seoul Korea; ^5^ Department of Preventive Medicine, College of Korean Medicine Kyung Hee University Seoul Korea

**Keywords:** ferroptosis, gene signature, head and neck squamous cell carcinoma, radiation therapy, statin

## Abstract

To enhance the efficacy of radiotherapy (RT) in human papillomavirus (HPV)‐negative head and neck squamous cell carcinoma (HNSCC), we explored targeting ferroptosis, a regulated cell death process. We developed a gene signature associated with ferroptosis using Cox proportional hazard modeling in HPV‐negative HNSCC patients who underwent RT. This ferroptosis‐related gene signature (FRGS) was a significant predictor of overall survival and recurrence‐free survival in HPV‐negative HNSCC patients who received RT. Subtype B of the FRGS, characterized by decreased expression of ferroptosis inducers [nuclear receptor coactivator 4 (*NCOA4*) and natural resistance‐associated macrophage protein 2 homolog/divalent metal transporter 1 (*NRAMP2/DMT1*)] and increased expression of suppressors [phospholipid hydroperoxide glutathione peroxidase (*GPX4*) and ferritin heavy chain (*FTH1*)], was associated with poorer prognosis, potentially indicating the inhibition of ferroptosis. Furthermore, our *in vitro* and *in vivo* studies demonstrated that treatment with statins, such as atorvastatin and simvastatin, induced ferroptosis and sensitized radioresistant HNSCC cells to irradiation, improving radiosensitivity and potentially enhancing the response to RT. Additionally, in xenograft models, the combination of statins and RT led to a significant reduction in tumor initiation. These findings provide valuable insights for enhancing treatment and improving prognosis in HPV‐negative HNSCC by targeting ferroptosis and utilizing statins to sensitize tumors to RT‐induced cell death.

## Graphical abstract

Radiosensitive head and neck squamous cell carcinoma (HNSCC) cells downregulate *GPX4* expression upon ionizing radiation (IR) exposure, inducing ferroptosis, whereas radioresistant HNSCC cells fail to do so. However, the combination of IR and statins can downregulate *GPX4* expression, thereby inducing ferroptosis in radioresistant HNSCC cells as well.

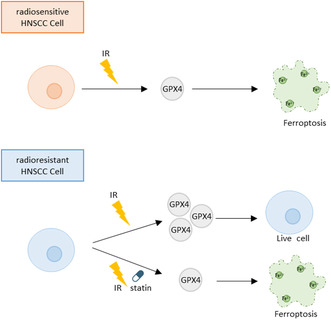

**Abbreviations**
BCCP: Bayesian compound covariate predictor
CAV1: caveolin‐1
CCLE: Cancer Cell Line Encyclopedia
CFA: colony formation assay
CRT: chemotherapy with radiotherapy
DMT1: divalent metal transporter 1
Fer‐1: ferrsotatin‐1
FHCRC: Fred Hutchison Cancer Research Center
FINs: ferroptosis inducers
FRGS: ferroptosis‐related gene signature
FRGs: ferroptosis‐related genes
FTH1: ferritin heavy chain 1
GPX4: glutathione peroxidase 4
HMG‐CoA: 3‐hydroxy‐3‐methyl‐glutaryl‐CoA
HMGCR: 3‐hydroxy‐3‐methylglutaryl‐CoA reductase
HNSCC: head and neck squamous cell carcinoma
HPV: human papillomavirus
HR: hazard ratio
IPP: isopentenyl pyrophosphate
IR: irradiation
KHU: Kyung Hee University
MVA: mevalonate
NCOA4: nuclear receptor coactivator 4
OS: overall survival
RCD: regulated cell death
RFS: recurrence‐free survival
RT: radiotherapy
SLC7A11: solute carrier family 7 member 11
TCGA: The Cancer Genome Atlas
TNBC: triple‐negative breast cancer

## 1 Introduction

Radiation therapy (RT) plays a pivotal role in the curative treatment of head and neck squamous cell carcinoma (HNSCC) [1]. Nevertheless, there is still ongoing research to improve tumor control and reduce radiation‐induced toxicity [2, 3]. While RT improves clinical, morphological, and functional outcomes for cancer patients, approximately 40% of HNSCC patients who undergo RT will experience local recurrence [4]. The establishment of precision medicine modalities is anticipated to extend survival and enhance the quality of life for advanced‐stage HNSCC patients with a poorer prognosis [5].

The classical risk factors for HNSCC include smoking and excessive alcohol consumption. Additionally, the human papillomavirus (HPV) has emerged as a valuable molecular factor for risk stratification and is associated with a favorable prognosis in HNSCC [6]. Previous studies have investigated the mechanisms of radiosensitivity in HNSCC, particularly focusing on the role of HPV. Previous studies have found that HPV‐positive HNSCC cell lines exhibit increased sensitivity to irradiation (IR) treatment compared to HPV‐negative cell lines [7, 8]. Understanding the reasons behind the resistance of HPV‐negative HNSCCs to IR may lead to improved therapeutic approaches.

Ferroptosis, a form of regulated cell death (RCD), is initiated with severe lipid peroxidation, which relies on reactive oxygen species (ROS) generation and iron availability [9, 10]. Unlike other forms of RCD, such as apoptosis, necroptosis, and pyroptosis, ferroptosis is characterized by disrupted cell membrane integrity and abnormal mitochondrial morphology [11]. It is considered an adaptive mechanism to eliminate malignant cells undergoing nutrient deficiency or damaged by infection or environmental stress [12]. Ferroptosis is regulated by various genes, including the ferroptosis‐suppressor genes (GPX4, SLC7A11, and FTH1), which have been identified as potential targets for cancer therapy [13].

The involvement of ferroptosis in cancer suppression has been widely recognized. The GPX4‐dependent surveillance system serves as the main monitoring and regulatory mechanism for ferroptosis in cancer cells [14]. Inhibition of ferroptosis has been shown to promote metastasis in gastric cancer by regulating GPX4 protein stability [15]. In triple‐negative breast cancer (TNBC), induction of ferroptosis by inhibiting GPX4 enhances anti‐tumor immunity, suggesting the potential of targeting ferroptosis in refractory TNBC [16]. Additionally, the overexpression of caveolin‐1 (CAV1) inhibits ferroptosis, leading to the development of an aggressive phenotype and a poorer prognosis [17]. Induction of ferroptosis by GPX4 inhibition has also been shown to sensitize head and neck cancer cells to cetuximab, enhancing treatment efficacy [18].

Statins, known as competitive inhibitors of 3‐hydroxy‐3‐methyl‐glutaryl‐CoA (HMG‐CoA) reductase, are commonly used to lower cholesterol levels. However, recent studies have discovered that statins possess anti‐tumor properties independent of their lipid‐lowering effects [19]. Zhang et al. [20] demonstrated that statin use reduces the risk of pancreatic cancer through a meta‐analysis including 170 000 pancreatic cancer patients in 26 studies. In colorectal cancer, a meta‐analysis of both randomized clinical trials and epidemiological studies revealed that statins may impact on outcomes by decreasing the invasiveness or metastatic properties [21]. These findings suggest that statins have potential as adjuvant cancer therapy and highlight the concept of drug repurposing in cancer treatment [22].

In this study, our aim was to identify effective prognostic biomarkers that can predict the outcomes of HPV‐negative HNSCC patients who undergo RT. Specifically, we investigated the potential of ferroptosis‐related genes as biomarkers that can influence radiosensitivity in these patients. By analyzing gene signatures, we obtained valuable insights into the role of ferroptosis in HNSCC and its potential as a therapeutic target. Furthermore, our research provides compelling evidence for considering statins as adjuvants to enhance the efficacy of RT in HNSCC. The anti‐cancer properties of statins, independent of their lipid‐lowering effects, suggest their potential as a readily available and well‐designed option for adjuvant cancer therapy.

## 2 Materials and methods

### 2.1 Patient and cohorts

The Cancer Genome Atlas (TCGA) cohort data were downloaded from the UCSC Cancer Genomics Browser (http://xena.ucsc.edu/) and used as the training set. 20 530 gene expression data plus clinical information for 520 HNSCC were downloaded from the TCGA database (https://gdc.cancer.gov/, assessed October 2020). In this study, 228 HPV‐negative patients who received RT were enrolled. Two independent datasets, a dataset from the Fred Hutchinson Cancer Research Center (FHCRC) (GSE41613) [23, 24] and a dataset from our institution (Kyung Hee University [KHU]), were used for independent validation. The FHCRC cohort data were downloaded from the National Center for Biotechnology Information Gene Expression Omnibus database (https://www.ncbi.nlm.nih.gov/geo/). The FHCRC cohort obtained 54 613 microarray gene expression data and clinical data of 97 HPV‐negative HNSCC patients. In particular, 53 of 97 HNSCC patients who received multi‐modality treatment were enrolled. Table S1 shows the clinical and pathological characteristics of each cohort. The TCGA, KHU, and FHCRC cohorts had 228, 39, and 53 patients with HNSCC, respectively. The KHU cohort was obtained from the Department of Otolaryngology‐Head and Neck Surgery, School of Medicine, Kyung Hee University. The experiments were undertaken with the understanding and written consent of each subject. This study was approved by and conducted in accordance with the policies set forth Institutional Animal Care and Use Committee of Kyung Hee University Medical Center (IACUC‐2023‐003). The study methodologies were approved by Kyung Hee University Medical Center (IRB: 2018‐05‐046‐12) and were conducted according to the principles of the Declaration of Helsinki. The methods have been previously described [25].

### 2.2 Cell culture

The human HNSCC cell lines CAL27 (RRID: CVCL_1107) and HSC4 (RRID: CVCL_1289) were purchased from the American Tissue Type Cell Collection (ATCC, Manassas, VA, USA) and the Japanese Collection of Research Bioresources (JCRB) Cell Bank (Osaka, Japan), respectively. The human HNSCC cell lines SNU1076 (RRID: CVCL_5006), SNU46 (RRID: CVCL_5063), and YD38 (RRID: CVCL_L083) were purchased from the Korean Cell Line Bank (KCLB, Seoul, Korea). Cells were cultured in RPMI‐1640 (HSC4, SNU1076, SNU46, and YD38) or DMEM (CAL27) medium supplemented with 10% heat‐inactivated fetal bovine serum (Hyclone, Logan, UT, USA) and 1% penicillin–streptomycin (Corning Inc., Corning, NY, USA). All cell lines were maintained at 37 °C in a humidified incubator containing 5% CO_2_. All cell lines were authenticated using short tandem repeat (STR) profiling in the past three years. A list of and the elated to online database have been made available for checking the authenticity of the cell lines in terms of the STR profile, as well as the correct name and description for each known cell line (https://www.cellosaurus.org/str‐search/). When thawing the initial cell cultures, all cell lines were supplied with a mycoplasma removal agent (MRA, #093050044; MP Biomedicals, Santa Ana, CA, USA) to eliminate any potential mycoplasma contamination.

### 2.3 Establishment of radioresistant cell lines

The radioresistant human HNSCC cell line, CAL27‐RR was established by applying repetitive various doses of X‐ray IR (2, 4, and 5 Gy). CAL27 cells were irradiated with 2 Gy per fraction (two times per week for 14.5 weeks), 4 Gy per fraction (one times per week for 3 weeks), and plus 5 Gy fraction until a final dose of 75 Gy. The irradiated cells were grown to 80–90% confluence and subcultured for continuous IR. The radioresistance phenotype was analyzed by colony formation assay. Parental human HNSCC cell line, CAL27 was maintained as control cells.

### 2.4 Development of gene signature

The list of ferroptosis‐related genes (FRGs) was downloaded from FerrDb (http://www.zhounan.org/ferrdb/index.html) [26] and contained 497 validated human FRGs. The interesting genes of FRGs were filtered by the Cox proportional hazard model based on the ‘survival’ r package, defining potential prognostic FRGs. The cutoff *P*‐value was 0.05, and 33 genes were selected as the FRG signature (FRGS). Among the 33 genes, 15 genes with a hazard ratio (HRs) > 1 were classified as high‐risk genes, and 18 genes with HRs < 1 were classified as low‐risk genes. Table S2 shows the genes included in the FRGS and their HRs and *P*‐values. We used data from 228 HPV‐negative HNSCC patients who received RT in TCGA to confirm that the FRGS could predict OS and RFS. Hierarchical clustering was performed using cluster 3.0 [27], and a heatmap was plotted using java treeviews [28]. Survival curves were generated and analyzed using the Kaplan–Meier method with the log‐rank test. Cox proportional hazard model and survival analyses were performed using r (http://www.r‐project.org).

### 2.5 Validation of the gene signature in independent cohorts

We validated the gene expression signature by classifying patients with HNSCC from independent cohorts using the Bayesian Compound Covariate Predictor (BCCP) algorithm [29]. Gene expression data from TCGA (training set) were grafted into the BCCP algorithm to generate discriminators. The BCCP algorithm is evaluated by the cross‐validation methods including leave‐one‐out cross‐validation, *k*‐fold validation, and 0.632+ bootstrap validation. Validation was conducted in the independent KHU and FHCRC cohorts. Predictive model construction and validation were performed using the BRB‐array tool (https://brb.nci.nih.gov/BRB‐ArrayTools/).

### 2.6 Chemicals

Atorvastatin and Ferrostatin‐1 (Fer‐1) were purchased from Sellekchem (S5715 and S7243, respectively; Selleckchem, Houston, TX, USA). Simvastatin was purchased from Sigma‐Aldrich (S6196; Sigma‐Aldrich, St.Louis, MO, USA).

### 2.7 Cell viability assay

YD38 and SNU1076 cells were seeded in 96‐well plates and treated with atorvastatin (10, 100, 1000, 10 000, and 100 000 nm) or simvastatin (10, 100, 1000, 10 000, and 100 000 nm). HSC4 and SNU46 cells were seeded in 96‐well plates and treated with Fer‐1 (100, 1000, 10 000, and 100 000 nm). 5 × 10^3^ cells/well were seeded in 96‐well plates. After 24 h of treatment with statins or Fer‐1, cell viability was measured according to the manufacturer's instructions (EZ‐cytox, DoGenBio, Seoul, Republic of Korea) and 10 μL of the reagent was added to each well. After 2 h of incubation in a CO_2_ incubator, the conversion of the reagent into chromogenic formazan was evaluated with a spectrophotometer at 570 nm.

### 2.8 Colony formation assay

HNSCC cell lines were trypsinized, diluted, and seeded into the triplicate 6‐well plate (200 and 300 cells/well, according to cell lines). After 24 h of incubation, cells were exposed to different doses of X‐ray (0, 2, 4, 6, and 8 Gy) using an XstrashI RS225 cabinet at room temperature with 195 kV/15 mA X‐rays, producing a dose rate of 1.6 Gy·min^−1^. Irradiated and untreated control cells were cultured for 8–16 days. In the case of statin‐treated experiments, after 20 h of incubation, cells were treated with statins (Atorvastatin and Simvastatin). After 2 h of treatment of statins, cells were exposed to different doses of X‐ray (0, 2, 4, 6, and 8 Gy). The next day, cells were treated with Ferrostatin‐1. Irradiated, statin‐treated, and untreated control cells were cultured for 8–17 days. The surviving cell‐derived colonies were stained with crystal violet solution (0.5% crystal violet in 50% methanol) and counted.

### 2.9 Western blot

Lysates from HNSCC cells were homogenized in RIPA buffer [1% Triton X‐100, 1% sodium deoxycholate, 0.1% sodium dodecyl sulfate (SDS), 150 mm NaCl, 50 mm Tris–HCl (pH 7.5), and 2 mm ethylenediamine tetra‐acetic acid (pH 8.0)] purchased from Biosesang (Seongnam‐si, Republic of Korea) containing a protease inhibitor cocktail. Protein concentration was then quantified using a Pierce Micro BCA Protein Assay Kit (ThermoFisher Scientific, Waltham, MA, USA), according to the manufacturer's protocol and equal amounts of protein, mixed with loading dye (5× SDS‐polyacrylamide gel electrophoresis loading buffer; iNtRON Biotechnology, Seongnam‐si, Republic of Korea), were added to each lane and resolved using an SDS‐polyacrylamide gel. Following electrophoresis, proteins were transferred to polyvinylidene difluoride membranes (Millipore, Burlington, MA, USA) and blocked for 1 h in 5% BSA and Tris‐buffered saline with 0.1% Tween‐20. These membranes were then treated with appropriate primary antibodies and incubated overnight at 4 °C. The following antibodies were used: [antibody list] anti‐NCOA4 (66849, rabbit monoclonal antibody; Cell Signaling Technology (CST), Danvers, MA, USA); anti‐DMT1/SLC11A2 (15083, rabbit monoclonal antibody; CST); anti‐GPX4 (52445, rabbit monoclonal antibody; CST); anti‐FTH1 (4393, rabbit monoclonal antibody; CST), and anti‐β‐actin (47778, mouse monoclonal antibody; Santa Cruz Biotechnology, Santa Cruz, CA, USA). The blots were then washed and treated with secondary antibody for 1 h at room temperature, and the protein‐antibody complexes were detected using enhanced chemiluminescence (RPN2232; GE Healthcare, Chicago, IL, USA) according to the manufacturer's protocol. The methods for western blot have been previously described [30].

### 2.10 Lipid peroxides measurement

Cells were seeded on 15 mm cover glass in 6‐well plates. The following day, either atorvastatin or simvastatin was treated in the cells, 2 h before IR. Fer‐1 was added 30 min after the IR. After 1, 6, 12, and 24 h of incubation, C11‐BODIPY (2 μm; ThermoFisher Scientific) was added into each well 30 min before measurements. Subsequently, cells were washed with PBS and observed using a Zeiss LSM710 confocal laser scanning microscope (Carl Zeiss AG, Oberkochen, Germany).

### 2.11 *In vivo* studies

Female BALB/c nude mice (5 weeks old, weighing 16–18 g) were purchased from Junbiotech (Hwaseong, Korea). This study was approved by and conducted in accordance with the policies set forth Institutional Animal Care and Use Committee of Kyung Hee University Medical Center (IACUC‐2023‐003). Mice were housed in a temperature and humidity‐controlled facility with a 12‐h light/dark cycle and provided with food and water *ad libitum*. All efforts were made to minimize animal suffering and reduce the number of animals used in this study. CAL27‐RR cells were cultured in DMEM medium plus 10% heat‐inactivated fetal bovine serum (Hyclone) and 1% penicillin–streptomycin (Corning Inc.). Cells were trypsinized, counted, and resuspended in PBS at a concentration of 2 × 10^7^ cells·mL^−1^ for injection into mice. CAL27‐RR cells with 2 × 10^6^ cells per 100 μL were injected subcutaneously into both thighs of each mouse, and the tumors were allowed to grow. The *n* numbers for tumor growth represent 8 tumors in total (with 4 mice, each having 2 tumors). After a day of injection of CAL27‐RR cells, atorvastatin (5 mg·kg^−1^) or simvastatin (2 mg·kg^−1^) were injected intraperitoneally for daily. After the 4th day of tumor injection, the tumors were treated with radiation at a dose of 2 Gy per session, a total of 5 treatments. The radiation schedule used in this study consisted of 2 Gy delivered daily for 5 days, resulting in a total dose of 10 Gy. The 5 × 2 Gy‐fractionated IR dose was used to mimic the protocol classically applied to patient for a week of treatment. The animals were weighed 2–3 days a week. For efficacy studies, tumors were measured every day by caliper. Efficacy studies were terminated about 18 days after initiation of IR. No mice met the criteria for euthanasia because of body weight loss nor exhibited adverse clinical signs during the studies.

### 2.12 Statistical analysis

Cox univariate and multivariate regression analyses were implemented to define the independent prognostic factor for OS. The predictive accuracy of the prognostic model for OS was evaluated by performing a time‐dependent ROC curve analysis. r software (version 4.21, R foundation, Vienna, Austria) was applied for all statistical analyses, and the ggplot2 [31] packages were used for graph visualization. Statistical significance was defined as *P* < 0.05, and all *P* values were two‐tailed.

## 3 Results

### 3.1 Development of ferroptosis‐related gene signature

**Fig. 1:**
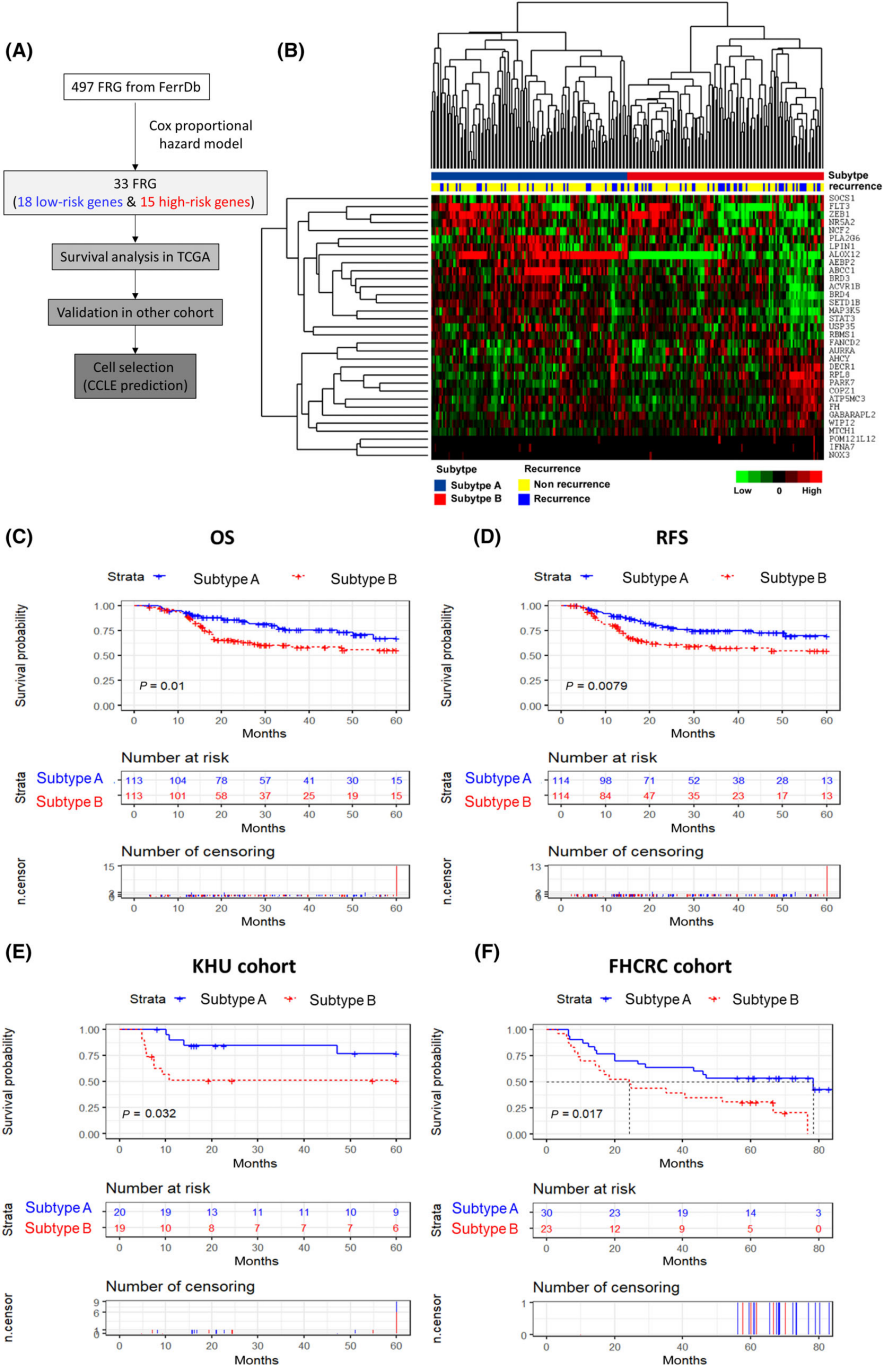
Development of ferroptosis‐related gene signature. (A) The schematic diagram for developing the ferroptosis‐related genes signature (FRGS) to predict patients' survival in head and neck squamous cell carcinoma (HNSCC). (B) Expression patterns of 33 FRGS with recurrence in the Cancer Genome Atlas (TCGA) cohort (*n* = 228). Green indicates low expression and red indicates high expression. In the recurrence panel, yellow indicates patients without recurrence and blue indicates patients with recurrence. Kaplan–Meier curve of the comparison between subtype A (*n* = 114) and subtype B (*n* = 114) of (C) overall survival (OS) and (D) recurrence‐free survival (RFS) in the TCGA cohort based on the FRGS subtype. The significance was calculated by a log‐rank test. Validation of ferroptosis‐related gene signature in independent cohorts. Kaplan–Meier curve of recurrence‐free survival in the (E) KHU and overall survival in the (F) FHCRC cohort (*n* = 39 and 53, respectively) based on the HNSCC subtype in the TCGA cohort. The significance of the Kaplan–Meier curve was calculated by the log‐rank test.

To identify an FRGS for HPV‐negative HNSCC, the 497 FRGs were applied to the Cox proportional hazard model. These genes were evaluated for their effect on RFS in the TCGA training cohort. The study design is illustrated using a flow diagram (Fig. 1A). The evaluation of these genes was carried out in the TCGA training cohort, where we identified 33 genes with *P*‐values < 0.05 as significant. Among the 33 genes acquired, 15 were associated with an increased risk of recurrence (HR > 1), while 18 were associated with a decreased risk of recurrence (HR < 1). The 33 genes collectively form the FRGS, as listed in Table S2.

To determine distinct patient subpopulations, hierarchical clustering analysis was performed using the FRGS as a predictor (Fig. 1B). HPV‐negative HNSCC patients who received RT were then categorized into two subtypes: FRGS subtype A (*n* = 114) and subtype B (*n* = 114). Notably, patients classified as subtype B FRGS exhibited poorer overall survival (OS) (Fig. 1C) and RFS (Fig. 1D). In the TCGA database for HPV‐positive HNSCC patients, there was no significant difference in OS and RFS between subtype A (*n* = 21) and subtype B (*n* = 22) (Fig. S1). These findings suggest that the FRGS may serve as a potential biomarker for predicting survival rates in HPV‐negative HNSCC.

Furthermore, comparing the expression levels of ferroptosis inducer genes (NCOA4, SLC7A11, TFRC, ALOX15, LPCAT3, and SLC11A2), subtype A demonstrated higher expression than subtype B (Fig. S2). Conversely, the expression of ferroptosis suppressor genes (GPX4 and FTH1) was induced in subtype B compared to subtype A. These findings collectively suggest that the differential expression of ferroptosis‐related genes between subtype A and subtype B indicates a potential inhibition of ferroptosis in subtype B.

### 3.2 Validation of the gene signature

The validation of the FRGS was conducted in two independent cohorts, KHU and FHCRC. This validation process confirmed the prognostic significance of FRGS. In the KHU cohort, patients who received RT were also classified as having subtype A or B based on the gene expression pattern of the FRGS, as depicted in the heatmap (Fig. S3A). Similar to the result from the TCGA cohort, the survival analysis of the KHU dataset revealed that patients with subtype B were more likely to have significantly shorter RFS and poorer prognosis than patients with subtype A (Fig. 1E). The FRGS was also validated in the FHCRC cohort using the method described above. Heatmap of FRGS genes was demonstrated using microarray data from the FHCRC cohort (Fig. S3B). Consistent with the KHU results, subtype B had a shorter OS than subtype A in the FHCRC cohort (Fig. 1F). The validation process in independent cohorts further confirmed the predictive value of the FRGS in determining patient outcomes.

### 3.3 The FRGS is an independent prognostic predictor in HNSCC

To assess the impact of the FRGS on RFS, we conducted univariate and multivariate Cox proportional regression analyses in three independent cohorts: TCGA, KHU, and FHCRC (*n* = 320), utilizing the available clinical data. The combination of the FRGS and the other clinicopathological factors (e.g., patient gender, age, smoking history, alcohol consumption, primary tumor, regional lymph node, and cancer stage) was used for the Cox proportional regression analysis. In both the univariate and multivariate analysis, FRGS subtype (subtype A vs. subtype B) was significantly associated with RFS (HR 2.050 and 1.973, 95% confidence interval 1.311–3.206 and 1.228–3.171; *P* = 0.00165 and 0.00497, respectively) (Table 1). These results highlight the substantial significance of the FRGS subtype as the only significant predictor in both univariate and multivariate analyses. Consequently, the FRGS demonstrates a high potential as an independent prognostic predictor. In addition, HNSCC patients' information between subtype A and subtype B were indicated in TCGA, KHU, and FHCRC cohort, respectively (Table S3). In the TCGA cohort, smoking demonstrated statistical significance, while in the FHCRC cohort, gender exhibited statistical significance. These divergent results may be attributed to several factors, such as differences in sample size, patient characteristics, or demographic distribution within each cohort.

**Table 1:** Univariate and multivariate Cox proportional hazard regression analysis of recurrence‐free survival in the TCGA, KHU, and FHCRC cohort (*n* = 320). CI, confidence interval; FRGS, ferroptosis‐related gene signature. Bold value indicates statistical significance by setting *P* < 0.05.

Variables	Univariate		Multivariate	
	HR (95% CI)	*P*	HR (95% CI)	*P*
FRGS subtype (subtype B)	2.050 (1.311–3.206)	**0.00165**	1.973 (1.228–3.171)	**0.00497**
Gender (male)	1.065 (0.616–1.840)	0.822	0.797 (0.431–1.475)	0.470
Age (≥ 60 years)	1.280 (0.817–2.004)	0.281	0.886 (0.555–1.415)	0.613
Smoking (yes)	1.025 (0.619–1.697)	0.925	1.284 (0.678–2.429)	0.443
Alcohol (yes)	1.327 (0.758–2.325)	0.323	1.224 (0.665–2.256)	0.516
Primary tumor (T3 and T4)	0.921 (0.545–1.556)	0.757	1.316 (0.506–3.424)	0.573
Regional lymph node (N+)	1.095 (0.696–1.724)	0.695	0.944 (0.560–1.592)	0.829
Stage (stage III and IV)	1.030 (0.514–2.065)	0.934	1.023 (0.277–3.778)	0.972

### 3.4 Selection of cell lines reflecting the characteristics of FRGS subtypes

Considering the practicality of utilizing established cancer cell lines as experimental models, we implemented the FRGS subtype to examine the gene expression data of HNSCC cell lines derived from the Cancer Cell Line Encyclopedia (CCLE) dataset. Based on their FRGS profiles, the analyzed 17 cell lines were subsequently categorized into two distinct subtypes (Fig. S4A). For further comparison of radiosensitivity between the predicted subtypes A and B, we conducted a colony formation assay (CFA) using the cell lines predicted to belong to each subtype by CCLE prediction (Fig. S4B). From subtype A, we selected the cell lines (HSC4, SNU46, and CAL27) with a survival rate below 5% at 8 Gy, while from subtype B, we chose the cell lines (SNU1076 and YD38) with a survival rate above 5% at 8 Gy. These selected cell lines were subsequently used for experimental validation. Based on the results of CFA, it was demonstrated that the subtype A cell lines exhibited higher radiosensitivity compared to the subtype B cell lines.

### 3.5 Statin treatment can modulate radiosensitivity and ferroptosis‐related proteins in HNSCC cells

Previous study demonstrated that pharmacological blockade of ferroptosis protects cancer cells from RT, which also appear to be radioresistant [32]. Also, statins have been reported to have anti‐cancer effects by modulating ferroptosis [33]. Building upon this knowledge, in this study, atorvastatin and simvastatin were used as ferroptosis inducers (FINs), while Fer‐1 was used as a ferroptosis inhibitor. To determine the concentration of these drugs, we initially examined the effects of statins on cell viability using an MTT assay. The death of HNSCC cells by statins was determined by quantifying viable cells in the absence or presence of statins at various concentrations after 24 h incubation. Statins decreased cell viability in a dose‐dependent manner (Fig. S5). Based on the growth inhibition curves, it was identified that the concentration Fer‐1 required for 50% inhibition of growth (IC50) for HSC4 cells was approximately 28.29 μm, and for SNU46 cells was approximately 31.33 μm. It was determined that the concentration of atorvastatin needed to achieve IC50 for SNU1076 cells was approximately 16.03 μm, while for YD38 cells it was approximately 23.85 μm. Furthermore, for simvastatin, the IC50 value for SNU1076 cells was found to be approximately 22.73 μm, and for YD38 cells it was approximately 23.85 μm.

**Fig. 2:**
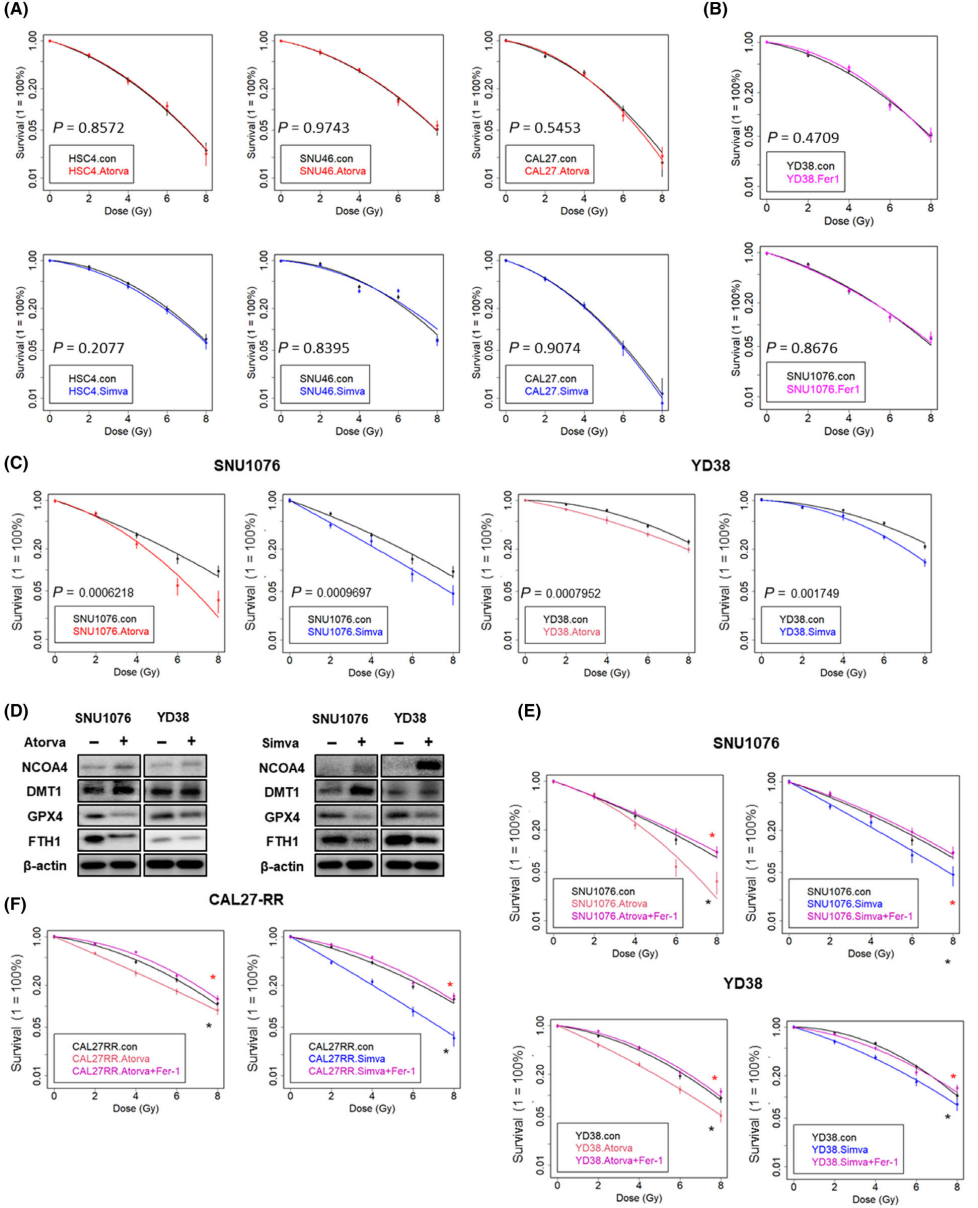
Statins modulate radiosensitivity and ferroptosis‐related proteins and Fer‐1 treatment abrogates statin‐mediated radiosensitization. (A) Colony formation assays (CFA) results showed no significant changes between control cells and Atorvastatin or Simvastatin treated cells in subtype A HNSCC cells (HSC4, SNU46, and CAL27). Seeded cells were exposed to ionizing radiation (IR) over the range of 0–8 Gy as indicated. *P*‐values indicate the significance of the differences at the 8 Gy dose in each experiment. Means (±SEM, standard error of mean) of at least three experiments are shown. (B) Subtype B HNSCC cells (YD38 and SNU1076) were treated with Fer‐1 and CFA was conducted. Atorva, atorvastatin; Fer‐1, ferrostatin‐1; Simva, simvastatin. (C) CFA showed that atorvastatin and simvastatin treatment both sensitized SNU1076 and YD38 subtype B HNSCC cells to IR. (D) The subtype B cell lines, after treatment with atorvastatin (10 μm) and simvastatin (5 μm), were evaluated for levels of ferroptosis‐related proteins. β‐Actin was included as an internal loading control. (E) Inhibition of ferroptosis induces resistance to ionizing radiation (IR) and abrogates statin‐mediated radiosensitization. CFA showed that ferrostatin‐1 (Fer‐1), an inhibitor of ferroptosis, abrogates statin‐mediated radiosensitization in radioresistant SNU1076 and YD38 subtype B cells. Cells were seeded and after 16 h, statins (atorvastatin or simvastatin) were added. Two hours after the addition of statins, IR was administered. Seeded cells were exposed to IR over the range of 0–8 Gy as indicated. *P*‐values indicate the significance of the differences at the 8 Gy dose in the experiment. Means (±SEM) of at three experiments are shown. Black asterisks indicate the presence of statistical significance between the control group and either atorvastatin or simvastatin treatment. Red asterisks represent statistical significance between atorvastatin or simvastatin treatment and the combination of statins and Fer‐1. *P < 0.05. (F) CFA showed that CAL27‐RR was sensitized to IR by statins. Furthermore, ferrostatin‐1 (Fer‐1) induces resistance to IR and abrogates statin‐mediated radiosensitization. Each point represents the mean of three replicates. *P*‐values are calculated at 8 Gy dose between control and statin‐treated, or between statin‐treated and a combination of statin and Fer‐1. CAL27‐RR cells were treated with 10 μm of atorvastatin or 5 μm of simvastatin for 2 h before IR (4 Gy). After 30 min, 5 μm of Fer‐1 was added to cells. Black asterisks indicate the presence of statistical significance between the control group and either atorvastatin or simvastatin treatment. Red asterisks represent statistical significance between atorvastatin or simvastatin treatment and the combination of statins and Fer‐1. *P < 0.05. All experiments were performed in triplicate. The data are presented as the mean ± standard error of the mean (SEM). Error bars represent the SEM. All CFA results were statistically analyzed using the *F*‐test statistical test.

To investigate whether statins and Fer‐1 treatment affect the response of RT, HNSCC cells were treated with statins or Fer‐1 and analyzed colony formation ability during IR. Relative radiosensitive subtype A cells (HSC4, SNU46, and CAL27) were treated with 5 μm of Fer‐1 during IR (Fig. S6A). These results demonstrated that Fer‐1 treatment reduced radiosensitivity in subtype A cells. When subtype A cells were treated with either Atorvastatin or Simvastatin, there was no observed change in radiosensitivity (Fig. 2A). Furthermore, western blot analyses were performed to assess whether 5 μm of Fer‐1 treatment could alter the expression of ferroptosis‐related proteins (Fig. S6B). In HSC4 and CAL27 cells, treatment with Fer‐1 decreased the expression of ferroptosis‐inducer proteins (NCOA4) and increased the expression of ferroptosis‐suppressor proteins (GPX4 and FTH1). The expression of DMT1, another ferroptosis‐inducer protein, did not show any significant changes upon Fer‐1 treatment. In the case of SNU46 cells, treatment with Fer‐1 to an increase in the expression of GPX4 and FTH1, while the expression of NCOA4 protein showed a decrease. However, there was an observed increase in the expression of DMT1 protein.

Subtype A (HSC4, SNU46, and CAL27) cells were treated with IR and either atorvastatin or simvastatin to observe changes in ferroptosis through lipid peroxidation using C11‐BODIPY staining (Fig. S7A). In all three cell lines, it was observed that 4 Gy IR at 24 h post‐treatment increased lipid peroxidation. In the case of HSC4 and CAL27 (excluding SNU46), the combination of 4 Gy IR and statin treatment further increased lipid peroxidation, while treatment with Fer‐1 resulted in a decrease. These findings suggest that subtype A cells exhibit increased ferroptosis when subjected to IR‐induced stress.

Furthermore, subtype B cell lines were treated with statins, atorvastatin and simvastatin. The treatment of both atorvastatin and simvastatin sensitized two subtype B cell lines (SNU1076 and YD38) to IR (Fig. 2C). However, CFA results showed no significant changed between control and Fer‐1 treatment group in subtype B cells (Fig. 2B). In SNU1076 cells treated with 10 μm of atorvastatin or 5 μm of simvastatin, the expression of NOCA4 and DMT1 was found to increase. Conversely, the expression of GPX4 and FTH1 was observed to decrease (Fig. 2D). These findings were consistent with the results seen in YD38 cells, where an increase in the expression of NCOA4 and DMT1 and a decrease in the expression of GPX4 and FTH1 were also observed. These findings indicate that statin treatment can influence the expression levels of ferroptosis‐related proteins.

Moreover, lipid peroxidation changes and ferroptosis were investigated in subtype B cell lines (YD38 and SNU1076), as well as in the radioresistant CAL27‐RR cell line, through C11‐BODIPY staining (Fig. S7B). In the case of SNU1076 cells, a decrease in lipid peroxidation was observed at 12 h after 4 Gy IR treatment. For YD38 and CAL27‐RR cells, a reduction in lipid peroxidation was observed at 24 h post 4 Gy IR treatment. These results indicate that subtype B cell lines and CAL27‐RR cells do not exhibit an increase in ferroptosis upon IR exposure.

### 3.6 Fer‐1 treatment abrogated statin‐induced sensitization to radiation in subtype B cell lines with relatively lower radiosensitivity

To estimate the ability to rescue radiosensitization by Fer‐1, cells treated with statins were subjected to IR (4 Gy) after 2 h and subsequently, after 16 h of incubation, Fer‐1 was added. The CFA was then conducted in subtype B cell lines (Fig. 2E). Indeed, treatment of Fer‐1 abrogated statin‐induced sensitization to IR. To verify whether the radiosensitization by Fer‐1 is actually mediated by ferroptosis, we performed CFA using Z‐VAD (an apoptosis inhibitor) and Necrostatin‐1 (a necrosis inhibitor) (Fig. S6C). Similar to the Fer‐1 treatment, after administering statins, IR was applied 2 h later, followed by treatment with Z‐VAD and Necrostatin‐1 in subtype B cells 30 min post‐IR. The CFA results from the two cell lines demonstrated that statin‐induced radiosensitivity was not rescued by either Z‐VAD or Necrostatin‐1. Therefore, the increased radiosensitivity induced by statins is associated with ferroptosis, rather than apoptosis or necrosis. To assess the ability of Fer‐1 to abrogate radiosensitization by regulation of ferroptosis‐related proteins, we conducted WB analysis to observe the changes in the expression of ferroptosis‐related proteins (Fig. S8A). The graphs represented the quantification of WB analyses (Fig. S8B). In SNU1076 cells, the expression of NCOA4 and DMT1 decreased, while the expression of GPX4 and FTH1 increased after exposure to IR. However, when cells were treated with both IR and either atorvastatin or simvastatin, the expression of NCOA4 and DMT1 increased compared to IR alone. Specifically, when atorvastatin or simvastatin was administered 2 h prior to IR, followed by Fer‐1 treatment 30 min after IR, there was no additional increase in the expression of NCOA4 and DMT1. Similarly, in YD38 cells, IR alone led to a decrease in DMT1 expression and an increase in GPX4 and FTH1 expression. The combination of IR and statins resulted in increased DMT1 expression and decreased GPX4 and FTH1 expression. However, there was no significant change in NCOA4 expression. The addition of Fer‐1 to the combination treatment reversed the changes in DMT1, GPX4, and FTH1 expression. These results suggest a potential resistance to ferroptosis in these cells.

**Fig. 3:**
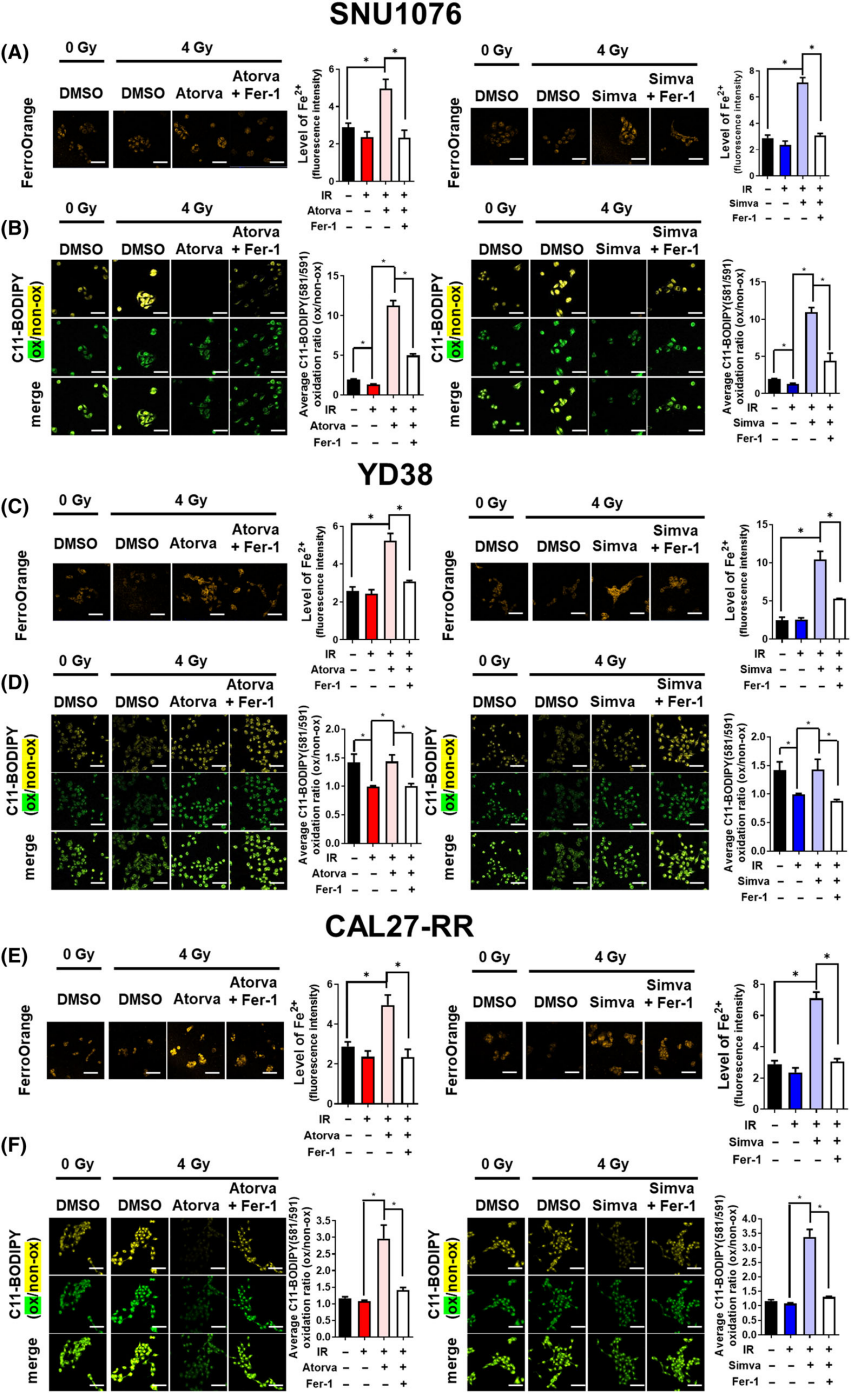
The combination of statins and ionizing radiation (IR) increased the levels of Fe^2+^ and lipid peroxidation and Fer‐1 treatment abrogated statin‐ and IR‐induced ferroptosis. (A) Representative confocal images of SNU1076 cells were stained with FerroOrange. The SNU1076 cells were treated with statin for 2 h, followed by IR exposure, and then Fer‐1 was added after an additional 30 min. After 6 h of Fer‐1 treatment, staining was performed, and the cells were analyzed using confocal microscopy. The quantitative graph represents levels of Fe^2+^ (fluorescence intensity). Scale bar = 100 μm. (B) Representative confocal images of SNU1076 cells stained with C11‐BODIPY. The SNU1076 cells were treated with statins, IR and Fer‐1 as described above. The quantitative graph represents the oxidation ratio, which is calculated by dividing the average intensity of the green channel (oxidized fluorescence) by the average intensity of the yellow channel (non‐oxidized fluorescence). Scale bar = 100 μm. (C) Representative confocal images of YD38 cells were stained with FerroOrange. Scale bar = 100 μm. (D) Representative confocal images of YD38 cells stained with C11‐BODIPY were captured. After 6 h of Fer‐1 treatment, staining was performed, and the cells were analyzed using confocal microscopy. The histogram represents the oxidation ratio. The significance was calculated using a two‐tailed test. *P < 0.05. Scale bar = 100 μm. (E) Representative confocal images of CAL27‐RR cells stained with FerroOrange. CAL27‐RR cells were treated with statins for 2 h, followed by IR exposure, and then Fer‐1 was added after an additional 30 min. Scale bar = 100 μm. (F) Representative confocal images of CAL27‐RR cells stained with C11‐BODIPY fluorescence in combination with IR (4 Gy) and statins (Atorvastatin; 10 μm and Simvastatin; 5 μm). The quantitative graph represents the oxidation ratio. Significance was calculated by a two‐tailed *t*‐test. The data are presented as the mean ± standard error of the mean (SEM). *P < 0.05. Scale bar = 100 μm. Atorva, atorvastatin; Fer‐1, ferrostatin‐1; Simva, simvastatin. All experiments were performed in triplicates. *P < 0.05.

Given the close association of NCOA4, DMT1, and FTH1 with iron homeostasis, we conducted Fe^2+^ measurements using FerroOrange staining in subtype B cells (SNU1076 and YD38) treated with statins, IR, and Fer‐1 (Fig. 3A,C). The results in both cell lines demonstrated that the combination of statins and IR led to an increase in Fe^2+^ levels compared to the control, whereas the addition of Fer‐1 subsequently reduced the increased Fe^2+^ levels. These findings suggest that statins may regulate ferroptosis in an iron‐dependent manner.

To assess the lipid peroxidation, indicator of ferroptosis, SNU1076, and YD38 cells were stained with C11‐BODIPY fluorescence. C11‐BODIPY fluorescence exhibits green color in the oxidized state and yellow in the non‐oxidized state. The ratio of oxidized to non‐oxidized C11‐BODIPY fluorescence, known as the oxidation ratio, reflects changes in lipid peroxidation. The increase in oxidation ratio indicates an elevated level of lipid peroxidation, which ultimately signifies the induction of ferroptosis. In SNU1076 cells, the impact of statin treatment followed by IR was evaluated at different time points (Fig. S7C). The most significant changes in lipid peroxidation were observed at 6 h after IR treatment. When SNU1076 cells were exposed to IR alone, the oxidation ratio decreased compared to non‐treated cells (Fig. 3B). However, the combination of either atorvastatin or simvastatin with IR led to a notable increase in the oxidation ratio. The addition of Fer‐1 effectively reduced the elevated oxidation ratio induced by the combination treatment.

Similarly, in YD38 cells, the most prominent changes in lipid peroxidation were also observed at 6 h after IR exposure (Fig. S7D). IR alone resulted in a decrease in the oxidation ratio compared to non‐treated cells (Fig. 3D). However, when combined with either atorvastatin or simvastatin, IR increased the oxidation ratio. The addition of Fer‐1 to the combination treatment effectively reduced the oxidation ratio. These findings suggest that the combination treatment of IR and statins has the potential to modulate the expression of ferroptosis‐related proteins and promote ferroptosis.

### 3.7 CAL27‐radioresistant cells showed reduced ferroptosis, while CAL27‐parent cells exhibited IR‐induced ferroptosis

To elucidate the association between resistance to RT and ferroptosis, we used CAL27‐radioresistant (CAL27‐RR) cell lines, established radioresistant cell lines. Treating CAL27‐P cells with IR significantly increased the expression of NCOA4 and decreased the expression of GPX4 (Fig. S9A). WB results showed that compared to CAL27‐P, CAL27‐RR exhibited lower basal levels of NCOA4 expression and significantly higher levels of GPX4 and FTH1 expression (Fig. S9B). The basal levels of DMT1 of CAL27‐RR were elevated than CAL27‐P. However, in the context of IR treatment, CAL27‐P cells showed a tendency to increase DMT1 expression. In contrast, CAL27‐RR cells showed significantly lower expression of DMT1 when exposed to 4 Gy of IR compared to the non‐irradiated condition. These results suggest that CAL27‐RR cells have lower basal expression levels of ferroptosis‐inducer proteins and higher basal levels of ferroptosis‐suppressor proteins, compared to CAL27‐P cells. In the case of DMT1, although CAL27‐RR exhibited higher basal expression levels, its expression decreased upon IR treatment, indicating the potential prevention of ferroptosis induction.

Furthermore, in both CAL27‐P and CAL27‐RR cells, after receiving a dose of 4 Gy of IR, staining was performed using C11‐BODIPY fluorescence (Fig. S9C). In CAL27‐P cells, upon exposure to IR, an increase in the oxidation ratio was observed, indicating an elevation in lipid peroxidation (Fig. S9D). This observed change suggests that in CAL27‐P cells, the treatment with IR resulted in the induction of ferroptosis. Conversely, in CAL27‐RR cells, the oxidation ratio decreased upon exposure to IR, indicating a decrease in ferroptosis.

### 3.8 Statins increase radiosensitivity of CAL27‐radioresistant cells by inducing ferroptosis

To investigate the regulatory capacity of statins in response to IR in CAL27‐RR cells, the cells were treated with statins followed by IR at different doses (2, 4, 6, and 8 Gy). Subsequently, the CFA was conducted to assess the outcomes (Fig. 2F). Both atorvastatin and simvastatin significantly enhanced the sensitivity of CAL27‐RR cells to IR. Moreover, the increased IR sensitivity observed with the combined treatment of IR and statins was subsequently reduced when Fer‐1 was added.

The results of WB showed the expression changes of ferroptosis‐related proteins in CAL27‐RR cells treated with statin and IR, respectively (Fig. S10A,B). Similar to the results in subtype B cell lines, CAL27‐RR cells irradiated with IR showed a significant decrease in the expression of NCOA4 and an increase in the expression of GPX4 and FTH1. This suggests that CAL27‐RR cells potentially inhibit ferroptosis when exposed to IR, indicating radioresistance. Although DMT1 showed no significant expression changes when only exposed to IR, its expression level significantly increased when IR and statin were combined. These results suggest that CAL27‐RR cells have the potential to inhibit ferroptosis by regulating ferroptosis‐related proteins under IR. However, the combined treatment of IR and statins induces ferroptosis in CAL27‐RR cells and ultimately increases radiosensitivity.

In CAL27‐RR cells, Fe^2+^ levels were assessed using FerroOrange staining (Fig. 3E). The confocal imaging results indicated a significant increase in Fe^2+^ levels in the cells treated with both statins and IR compared to the control group. When Fer‐1 was added, it led to a reduction in the elevated Fe^2+^ levels.

To determine the time at which changes in lipid peroxidation are observed in CAL27‐RR cells, we compared the oxidation ratio at 1, 6, 12, and 24 h after IR (Fig. S10C). The results indicated that CAL27‐RR cells exhibited the most significant difference at 24 h. To further investigate CAL27‐RR cells, additional confocal experiments were conducted at 24 h after IR exposure (Fig. 3F). When CAL27‐RR cells were treated with 4 Gy of IR in combination with the two statins, a significant increase in oxidation ratio was observed. This indicates that lipid peroxidation is significantly enhanced when statin treatment is combined with IR. Furthermore, it was observed that the increased ferroptosis induced by IR and statins was attenuated upon the addition of Fer‐1.

### 3.9 Statins enhance the radiation‐induced regression of tumors in xenograft models

**Fig. 4:**
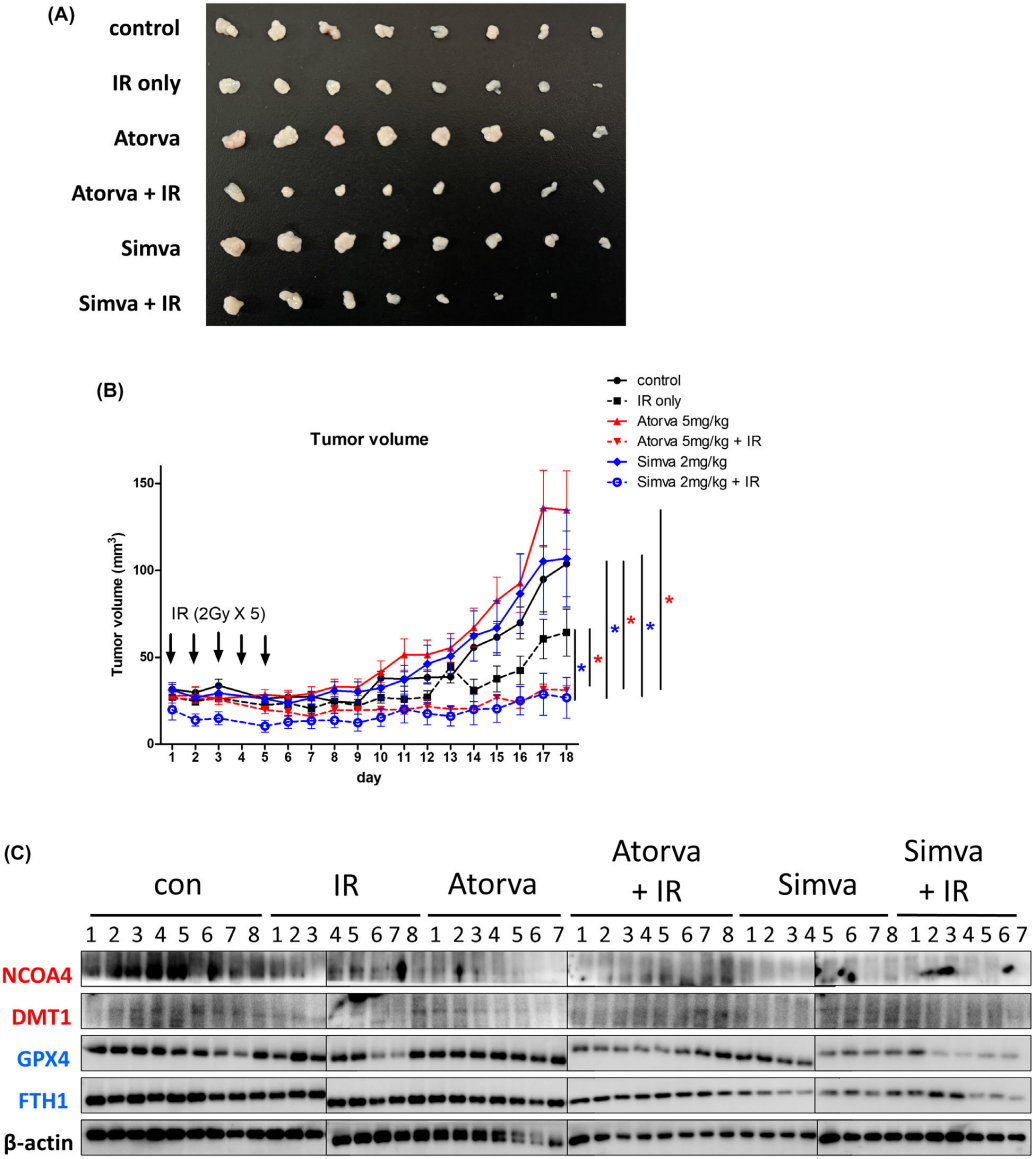
Statins increase the efficiency of radiation therapy in CAL27‐RR xenograft mouse model. (A) Photos of the excised tumors on day 18. (B) The graph presents the changes in tumor volume (mm^3^) in the control (saline), ionizing radiation (IR) only, atorvastatin, and atorvastatin + IR. Tumor volume was measured every day. Also, the tumor growth curve shows an elevation of tumor size during 18 days in the control, IR only, simvastatin, and simvastatin + IR. Statistical significance was calculated using the Mann–Whitney *U* test. The red asterisk indicates *P* < 0.05 in control vs atorvastatin + IR, IR only vs atorvastatin + IR, or atorvastatin vs atorvastatin + IR. The blue asterisk indicates *P* < 0.05 in control vs simvastatin + IR, IR only vs simvastatin + IR, or simvastatin vs simvastatin + IR. Error bars, standard error of mean (SEM). (C) The protein levels of ferroptosis‐related proteins, NCOA4, DMT1, GPX4, and FTH1, were analyzed by western blot in mouse tumor samples. The number of tumors per group ranged from 7 to 8. All experiments were performed in triplicate.

Based on significant *in vitro* and clinical data, a preclinical evaluation was conducted using an *in vivo* model with CAL27‐RR cells implanted as xenograft tumors in BALB/c nude mice. The mice were randomly assigned to four treatment groups: control, IR only, statin only, and combination of statin and IR (Fig. S11A). The photograph showed the excised tumors on day 18 (Fig. 4A). Tumor growth was monitored for up to 18 days after IR treatment to assess the combined effect of atorvastatin and IR on tumor growth (Fig. 4B). Remarkably, the mice treated with both atorvastatin and IR exhibited a significant regression in tumor volume compared to the single treatment groups receiving atorvastatin or IR alone. Additionally, a similar study was conducted using simvastatin to evaluate its combined effect with IR on tumor growth *in vivo*. The methods employed were the same as described above. The combination of simvastatin and IR showed significant regression, compared with the single treatment group with simvastatin or IR alone. The total body weights of the mice remained unchanged throughout the 18‐day experiment in both the atorvastatin and simvastatin experiments (Fig. S11B). This demonstrates that the combination of either atorvastatin or simvastatin with IR did not lead to any significant changes in the overall weight of the mice, further supporting the safety and tolerability of these treatment approaches. We compared the expression of ferroptosis‐related proteins across different groups (control, IR only, statin only, and the combination of statin and IR) using WB (Fig. 4C and Fig. S11C). The results showed a significant increase in the expression of DMT1 in the combination of statin and IR group compared to the control group, while the expression of GPX4 significantly decreased. These findings highlight that the combination of statins and IR significantly suppressed tumor growth compared to the control, IR‐only, or statin‐only groups. These results demonstrated that statins significantly enhanced the radiosensitivity of HNSCC *in vivo*.

## 4 Discussion

To enhance the efficacy of RT, current efforts have mainly focused on combining chemotherapy with radiotherapy (CRT) to sensitize tumor cells to IR [34]. However, clinical trials CRT have shown limited improvements in efficacy [35, 36]. Therefore, there is a persistent need for research into novel therapeutic approaches that target the unique biological characteristics of cancer cells.

Ferroptosis, an iron‐dependent regulated cell death process, has emerged as a potential therapeutic target for enhancing the effectiveness of RT [14]. In this study, we conducted a gene signature analysis to identify ferroptosis‐related genes associated with the RFS of HPV‐negative HNSCC patients who received RT. The analysis revealed that FRGS consists of 33 FRGs that can predict the OS and RFS of HNSCC patients who received RT. The training, validation and external independent cohorts proved that the FRGS was robust and had a high prediction ability.

Furthermore, we performed *in vitro* studies using HNSCC cells and demonstrated that statins, commonly used for hypercholesterolemia, can sensitize radioresistant HNSCC cells to IR by inducing ferroptosis. In previous studies, IR induces ferroptosis in cancer cells. However, IR also induces the expression of ferroptosis inhibitors, including solute carrier family 7 member 11 (SLC7A11) and GPX4, as an adaptive response [32]. Our study elucidates subtype B shows lower radiosensitivity than subtype A, which can be attributed to the higher expression of ferroptosis‐suppressor proteins and the generally lower expression of ferroptosis‐inducer proteins in subtype B, suggesting a potential role of ferroptosis inhibition in conferring radioresistance. In addition, statins effectively increase ferroptosis, which is suppressed by IR, thereby making subtype B sensitive to IR. We propose statin, a FINs, as a therapeutic method to increase the efficiency of RT by targeting ferroptosis in HNSCC.

Statins, which block the synthesis of mevalonate by HMGCR, are treated for hypercholesterolemia. A previous study reported that the anti‐cancer effects of statins, independently achieve a reduction in cholesterol synthesis in hypercholemia [37]. A major hurdle in the repurposing of statins as anti‐cancer drugs is the identification of adaptive features that reflect the radioresistance of cancer cells. Identifying the molecular determinants (i.e. biomarkers) that reflect adaptive features will help improve clinical practice.

Ferroptosis has been recognized as a critical RCD triggered by RT. Previous studies revealed that IR induces ferroptosis and that ferroptosis represents an important part of RT‐mediated anti‐cancer effects [38]. Indeed, cancer cells have been found to utilize the ferroptosis surveillance system to evade IR‐induced ferroptosis. This surveillance system comprises two major mechanisms, known as GPX4‐dependent and FSP1‐dependent pathways. Our WB results illustrated that IR increased the expression of GPX4 than non‐treated in subtype B cell lines, indicating subtype B cell lines potentially inhibited ferroptosis. Also, in an established radioresistant cell (CAL27‐RR), GPX4 was upregulated under IR‐only treatment compared to the parent cell. Considering these results, ferroptosis inhibition by IR is due to an increase of GPX4 and consequently causes radioresistance. GPX4 is a key ferroptosis‐related gene. GPX4 normally removes the dangerous products of iron‐dependent lipid peroxidation, inhibiting ferroptosis. The loss of activity of GPX4 results in an overwhelming accumulation of lethal lipid peroxides [9]. The overexpression of GPX4 constitutes the one of major surveillance systems to defend against ferroptosis in cancer cells [39].

Furthermore, iron homeostasis is crucial in regulating ferroptosis. DMT1/SLC11A2 releases Fe^2+^ into the cytoplasm and Fe^2+^ is confirmed by the ferritin, the main iron storage protein [40]. Ferritin heavy chain 1 (FTH1) oxidizes Fe^2+^ into Fe^3+^ and stores iron [41] The nuclear receptor coactivator 4 (NCOA4) recognized the ferritin. In case of deficiency of iron, NCOA4 localized ferritin to lysosomes and then ferritin degraded [42]. Failure of iron homeostasis leads to the Fenton reaction [40]. The Fenton reaction increases ROS, consequently causing ferroptosis [43]. The observed changes in DMT1, NCOA4, and FTH1 in our WB results suggest that the alteration in ferroptosis induced by IR or statin treatment in HPV‐negative HNSCC cells could be attributed to an imbalance in iron homeostasis.

Moreover, GPX4 is known to be activated by isopentenyl pyrophosphate (IPP), an intermediate of the MVA pathway. Statins are well‐known inhibitors of the HMGCR enzyme, which is involved in the initial steps of the MVA pathway [44]. Therefore, it is anticipated that the regulation of ferroptosis by statins is likely to be associated with the MVA pathway and is also presumed to have a correlation with GPX4. Atorvastatin induces ferroptosis by suppressing the intracellular anti‐oxidative system, Nrf2‐xCT/GPX4 pathway, resulting in lethal lipid peroxidation [45]. Simvastatin could inhibit HMGCR to modulate the mevalonate (MVA) pathway and deactivate GPX4 [46]. IR‐mediated induction of GPX4 reversed under treatment of statins, Atorvastatin and Simvastatin. Fer‐1 attenuated ferroptosis by targeting at SLC7A11/GPX4 pathway [47, 48]. Adding Fer‐1 restored the reduced expression of GPX4 in cells treated with IR and statins. The data presented here have important clinical implications for statins as anti‐cancer agents. However, therapeutic induction of ferroptosis must be carefully assessed to identify suitable compounds, doses, and schedules, as well as appropriate neoplastic indications, due to the potential detrimental side effects of FINs on normal bone marrow cells and various tissues [49]. Therefore, it is essential to thoroughly evaluate strategies for targeted ferroptosis induction to ensure optimal outcomes.

Furthermore, the results from our *in vivo* experiments provide valuable insights into the potential therapeutic application of statins in combination with IR for the treatment of HNSCC. By using CAL27‐RR xenograft tumors in mice, we investigated the effects of both atorvastatin and simvastatin in combination with IR on tumor growth. Our findings demonstrated that the combination of either atorvastatin or simvastatin with IR led to a significant regression in tumor volume compared to the single treatment groups receiving statin alone or IR alone. This indicates that statins can enhance the radiosensitivity of HNSCC *in vivo*, suggesting a synergistic effect between the two treatment modalities. Overall, our results support the potential therapeutic benefits of combining statins with IR for the treatment of HNSCC. The combination therapy demonstrated significant tumor regression without compromising the overall well‐being of the mice. These findings provide a foundation for future studies exploring the underlying mechanisms and optimization of the treatment regimen to maximize its effectiveness in clinical settings.

## 5 Conclusions

The FRGS was identified as a potential indicator of survival rate in HPV‐negative HNSCC, highlighting the importance of ferroptosis as a targetable mechanism to enhance the efficacy of RT and improve treatment outcomes. Furthermore, our research suggests that statins, acting as ferroptosis inducers, hold promise as anti‐cancer agents for sensitizing cancer cells to IR, thereby offering potential benefits for HPV‐negative HNSCC patients.

## Conflict of interest

The authors declare no conflict of interest.

## Author contributions

JKN, and Y‐GE contributed to conceptualization; JKN, JWL, YCL, and SRW contributed to data curation; JKN, MKL, and SIK contributed to formal analysis; JKN, SM, MK, YCL, and S‐GK contributed to methodology; S‐GK and Y‐GE contributed to project administration; Y‐GE contributed to supervision; JKN and SIK contributed to validation; YL and MB contributed to visualization; JKN contributed to roles/writing – original draft; SRW and Y‐GE contributed to writing – review & editing.

### Peer review

The peer review history for this article is available at https://www.webofscience.com/api/gateway/wos/peer‐review/10.1002/1878‐0261.13720.

## Supporting information


**Fig. S1.** Ferroptosis‐related gene signature (FRGS) does not show predictive power for prognosis in HPV‐positive HNSCC cohort.
**Fig. S2.** The expression of ferroptosis‐related genes is elevated in subtype A than subtype B.
**Fig. S3.** Validation of the ferroptosis‐related gene signature was conducted in additional cohorts to ensure its robustness and reliability.
**Fig. S4.** Sensitivity of HNSCC cell lines to radiation treatment.
**Fig. S5.** Statins exert a regulatory effect on the sensitivity of cells to radiation and the expression of proteins involved in ferroptosis.
**Fig. S6.** Ferroptosis is related with radioresistance in HNSCC cells.
**Fig. S7.** Lipid peroxidation changes upon radiation, statin, or Fer‐1 treatments.
**Fig. S8.** The application of Fer‐1 counteracts the radiosensitizing effects of statins in subtype B cells, specifically in SNU1076 and YD38 cells.
**Fig. S9.** CAL27‐RR cells showed inhibited ferroptosis than CAL27‐P.
**Fig. S10.** Statins modulated the protein levels of ferroptosis‐related proteins and induced significant changes in lipid peroxidation in CAL27‐RR cells.
**Fig. S11.** Statins enhance the efficacy of radiation therapy in a xenograft mouse model of CAL27‐RR.

**Table S1.** Head and neck squamous cell carcinoma patient's information of TCGA, KHU, and FHCRC cohort. All patients have HPV‐negative cancer and received RT.

**Table S2.** The list of 33 ferroptosis‐related genes comprising FRGS.

**Table S3.** Patients' information between subtype A and subtype B were listed in cohorts from TCGA, KHU, and FHCRC.

## Data Availability

The data generated in this study are available within the article and its Supporting Information files. Expression profile data analyzed in this study were obtained from Gene Expression Omnibus (GEO) at GSE41613. Raw data for RNA‐seq and microarray data are available in the public open access repository (NCBI database).
