# Supplementary material for: Targeting ferroptosis for improved radiotherapy outcomes in HPV‐negative head and neck squamous cell carcinoma
Source: Mol Oncol. 2024 Sep 19;19(2):540–57. doi: 10.1002/1878-0261.13720 (PMC11792990; doi:10.1002/1878-0261.13720)
Supplement: Supplementary file 1 — Fig. S1. Ferroptosis‐related gene signature (FRGS) does not show predictive power for prognosis in HPV‐positive HNSCC cohort. Fig. S2. The expression of ferroptosis‐related genes is elevated in subtype A than subtype B. Fig. S3. Validation of the ferroptosis‐related gene signature was conducted in additional cohorts to ensure its robustness and reliability. Fig. S4. Sensitivity of HNSCC cell lines to radiation treatment. Fig. S5. Statins exert a regulatory effect on the sensitivity of cells to radiation and the expression of proteins involved in ferroptosis. Fig. S6. Ferroptosis is related with radioresistance in HNSCC cells. Fig. S7. Lipid peroxidation changes upon radiation, statin, or Fer‐1 treatments. Fig. S8. The application of Fer‐1 counteracts the radiosensitizing effects of statins in subtype B cells, specifically in SNU1076 and YD38 cells. Fig. S9. CAL27‐RR cells showed inhibited ferroptosis than CAL27‐P. Fig. S10. Statins modulated the protein levels of ferroptosis‐related proteins and induced significant changes in lipid peroxidation in CAL27‐RR cells. Fig. S11. Statins enhance the efficacy of radiation therapy in a xenograft mouse model of CAL27‐RR. [file MOL2-19-540-s001.zip › mol213720-sup-0001-Supinfo.docx]

**Supporting Information**

**Supplementary figure 1**. Kaplan-Meier curve of the comparison between subtype A (n=21) and subtype B (n=22) of (A) overall survival (OS) and (B) recurrence-free survival (RFS) in the TCGA HPV-positive HNSCC cohort of patients who received RT, based on the FRGS subtype. The significance was calculated by a log-rank test.

**Supplementary figure 2.** The expression of ferroptosis-related genes is elevated in subtype A than subtype B. Ferroptosis-inducer and suppressor genes list downloaded from FerrDb. Five genes in the upper 2 lines of the figure (from NCOA4 to SLC11A2) were ferroptosis-inducer genes. GPX4 and FTH1 were ferroptosis-suppressor genes. Significance was calculated by a two-tailed t-test.

**Supplementary figure 3.** Validation of the Ferroptosis-related gene signature was conducted in additional cohorts to ensure its robustness and reliability. Heatmap analysis of FRGS in the (A) KHU and (B) FHCRC cohorts.

**Supplementary figure 4.** Sensitivity of HNSCC cell lines to radiation treatment. (A) FRGS subtypes in HNSCC cell lines (n = 17 in CCLE data set). HNSCC cell lines were stratified according to Bayesian compound covariate predictor probability from the FRGS predictor. Blue indicates subtype A and orange indicates subtype B. The cell lines highlighted in bold in the table represent the cell lines used in subsequent experiments for each subtype. (B) Log-linear plot demonstrating the relative sensitivity of the 5 subtype A HNSCC cell lines (HSC4, SNU1076, SNU46, CAL27, and YD38) to IR of 0 to 8Gy as determined by a colony formation assay. Each point represents the mean of 5 replicates. Significance was calculated by a two-tailed t-test. **p<0.01 for HSC4 or CAL27 vs. other cells.

**Supplementary figure 5.** Statins exert a regulatory effect on the sensitivity of cells to radiation and the expression of proteins involved in ferroptosis. MTT assays were performed in non-treated, statin-treated and ferrostatin-1 (Fer-1)-treated HNSCC cells.

**Supplementary figure 6.** Ferroptosis is related with radioresistance in HNSCC cells. (A) Colony formation assays showed that Fer-1 treatment increased the radioresistance of three subtype A HNSCC cells (HSC4, SNU46, and CAL27). Seeded cells were exposed to ionizing radiation (IR) over the range of 0 to 8Gy as indicated. P-values indicate the significance of the differences at the 8Gy dose in each experiment. Means (±SEM) of at least 3 experiments are shown. (B) Ferroptosis-related protein levels in subtype A cell lines were determined using a western blot. Cells were seeded and after 6h, Fer-1 (5μM) was added. After 24h incubation cells were harvested. β-actin was included as an internal loading control. (C) Colony formation assays revealed that the use of Z-vad (an apoptosis inhibitor) and Necrostatin-1 (a necrosis inhibitor) did not counteract the radiosensitization effects of statins in radioresistant SNU1076 and YD38 subtype B cells. Cells were seeded and after 16h, statins (atorvastatin or simvastatin) were added. Two hours after the addition of statins, IR was administered. Seeded cells were exposed to IR over the range of 0 to 8 Gy as indicated. Z-VAD and Necrostatin-1 were administered two hours after the IR treatment. P-values indicate the significance of the differences at the 8Gy dose in the experiment. Means (±SEM) of at least 3 experiments are shown. Black asterisks indicate the presence of statistical significance between the control group and either atorvastatin or simvastatin treatment. *p<0.05.

**Supplementary figure 7.** Lipid peroxidation changes upon radiation, statin, or Fer-1 treatments. (A) Lipid peroxidation changes in subtype A (HSC4, SNU46, CAL27) cells. The cells were treated with statins 2 hours before IR exposure, followed by the addition of Fer-1 after 30 minutes. Lipid peroxidation changes were observed at 6 hours, 12 hours, and 24 hours after IR exposure using C11-BODIPY staining. The oxidation ratio, calculated by dividing the average intensity of the green channel (oxidized fluorescence) by the average intensity of the yellow channel (non-oxidized fluorescence), is shown in the quantitative graph. (B) Lipid peroxidation changes in subtype B (YD38, SNU1076, CAL27-RR) cells. The cells were treated with statins and Fer-1 using the same method as described above. Lipid peroxidation changes were observed at 6 hours, 12 hours, and 24 hours after IR exposure using C11-BODIPY staining. The statistical significance was determined using a t-test, with *p<0.05 indicating significance. (C) After being treated with statins, the SNU1076 cells were subjected to IR after 2h, followed by the addition of Fer-1 after 30 min. Subsequently, changes in lipid peroxidation were observed at 6h and 24h using C11-BODIPY staining. The quantitative graph represents the oxidation ratio, which is calculated by dividing the average intensity of the green channel (oxidized fluorescence) by the average intensity of the yellow channel (non-oxidized fluorescence). (D) YD38 cells were treated with statins and exposed to IR after 2h. Fer-1 was then added after 30min. Changes in lipid peroxidation were subsequently observed at 6h and 24h using C11-BODIPY staining.

**Supplementary figure 8.** The application of Fer-1 counteracts the radiosensitizing effects of statins in subtype B cells, specifically in SNU1076 and YD38 cells. (A) Subtype B cells were treated with statins (Atorvastatin; 10μM and Simvastatin; 5μM) for 2h before IR (4 Gy). After 6h of IR, 5μM of Fer-1 was added to cells. The protein levels of ferroptosis-related proteins (inducer genes of ferroptosis, NCOA4 and DMT1; suppressor genes of ferroptosis, GPX4 and FTH1) were analyzed by western blot. Atorva, atorvastatin; Simva, simvastatin; Fer-1, ferrostatin-1. (B) The histograms represent the relative intensity of the protein band of NCOA4, DMT1, GPX4, and FTH1. All protein levels were normalized to the β-actin as a control. Error bars, SEM. *p<0.05, respectively.

**Supplementary figure 9.** CAL27-RR cells showed inhibited ferroptosis than CAL27-P. (A) Both CAL27-P and CAL27-RR cells were exposed to different doses of IR (0, 4, and 8Gy). Western blot of ferroptosis-related genes in head and neck squamous cell carcinoma cells 24h after IR. β-actin was included as an internal loading control. (B) The graph represents the quantification of Western blot results. The expression levels were normalized to the β-actin signal. Data are presented as mean ± standard deviation (SEM) from three independent experiments. *p<0.05. (C) Representative confocal images of CAL27-P and CAL27-RR cells showing an increase in C11-BODIPY fluorescence 6h after IR. (D) Mean C11-BODIPY(581/591) oxidation ratio for three biological replicates. A single asterisk indicates p<0.05 for a t-test for the difference of means between two conditions for each given comparison. Error bars, SEM.

**Supplementary figure 10.** Statins modulated the protein levels of ferroptosis-related proteins and induced significant changes in lipid peroxidation in CAL27-RR cells. (A) The protein levels of ferroptosis-related proteins, NCOA4, DMT1, GPX4, and FTH1, were analyzed by western blot. (B) The quantitative graph of expression of ferroptosis-related proteins from three independent experiments in CAL27-RR cells. (C) After being treated with statins, the CAL27-RR cells were subjected to IR after 2h, followed by the addition of Fer-1 after 30 min. Subsequently, changes in lipid peroxidation were observed at 1, 2, 6, and 24h using C11-BODIPY staining. The quantitative graph represents the oxidation ratio.

**Supplementary figure 11**. Statins enhance the efficacy of radiation therapy in a xenograft mouse model of CAL27-RR. (A) Experimental schedule for the in vivo study. After 7-day acclimation period, tumors were injected into both thighs of the mice. The following day, statin treatment was administered via intraperitoneal injection for daily. IR treatment was given from day 1 to 5, and on day 18, the mice were sacrificed for analysis. (B) The graph shows the changes in mouse body weight throughout the study. (C) The quantitative graph of expression of ferroptosis-related proteins from mouse tumors (n=7~8 per group).

**Supplementary table 1.** Head and neck squamous cell carcinoma patient’s information of TCGA, KHU, and FHCRC cohort. All patients have HPV-negative cancer and received RT.

**Supplementary table 2.** The list of 33 Ferroptosis-related genes comprising FRGS.

**Supplementary table 3.** Patients' information between subtype A and subtype B were listed in cohorts from TCGA, KHU, and FHCRC.
