# Supplementary figures and images for: Targeting ferroptosis for improved radiotherapy outcomes in HPV‐negative head and neck squamous cell carcinoma
Source: Mol Oncol. 2024 Sep 19;19(2):540–57. doi: 10.1002/1878-0261.13720 (PMC11792990; doi:10.1002/1878-0261.13720)

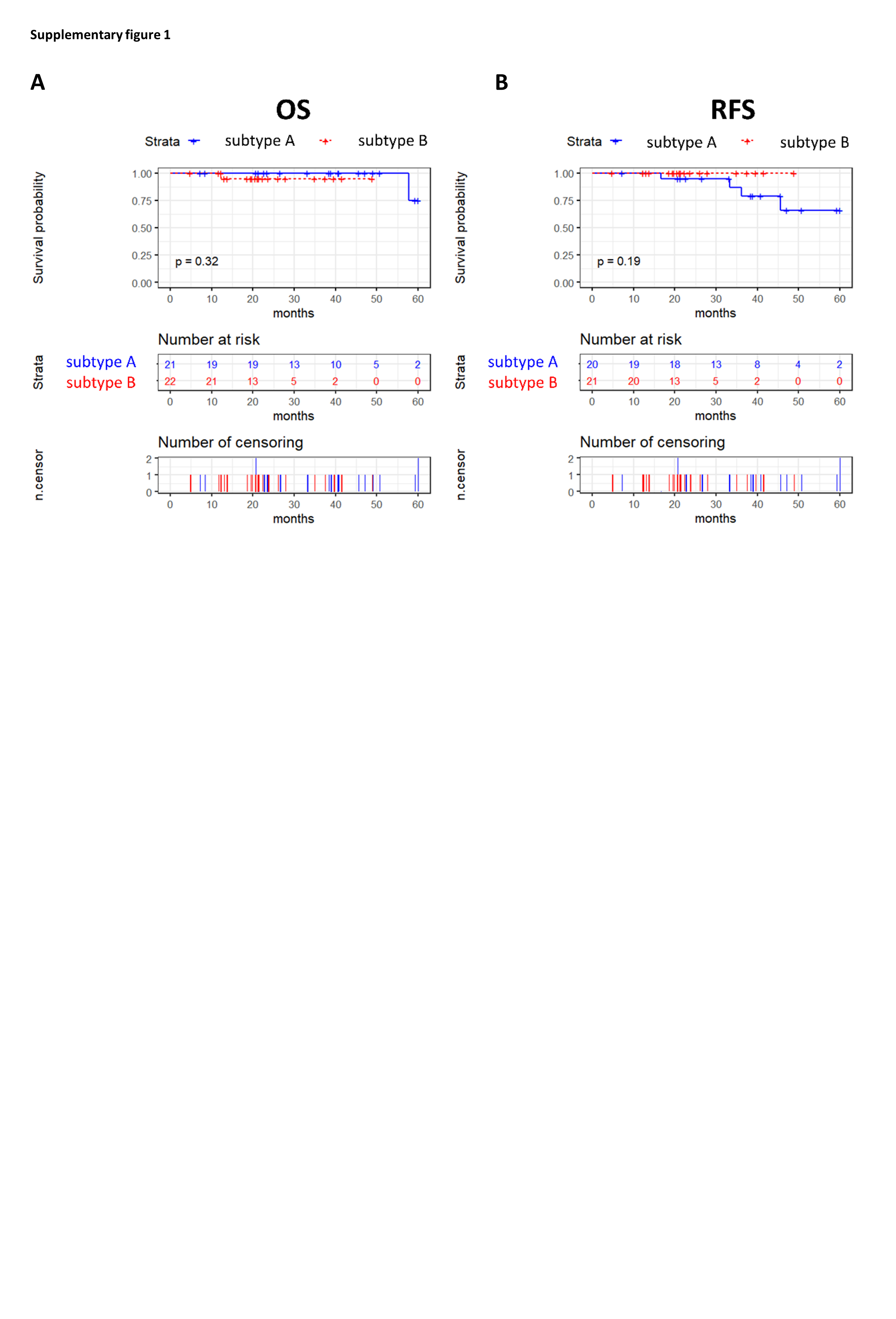

Supplement: Supplementary file 1 — Fig. S1. Ferroptosis‐related gene signature (FRGS) does not show predictive power for prognosis in HPV‐positive HNSCC cohort. Fig. S2. The expression of ferroptosis‐related genes is elevated in subtype A than subtype B. Fig. S3. Validation of the ferroptosis‐related gene signature was conducted in additional cohorts to ensure its robustness and reliability. Fig. S4. Sensitivity of HNSCC cell lines to radiation treatment. Fig. S5. Statins exert a regulatory effect on the sensitivity of cells to radiation and the expression of proteins involved in ferroptosis. Fig. S6. Ferroptosis is related with radioresistance in HNSCC cells. Fig. S7. Lipid peroxidation changes upon radiation, statin, or Fer‐1 treatments. Fig. S8. The application of Fer‐1 counteracts the radiosensitizing effects of statins in subtype B cells, specifically in SNU1076 and YD38 cells. Fig. S9. CAL27‐RR cells showed inhibited ferroptosis than CAL27‐P. Fig. S10. Statins modulated the protein levels of ferroptosis‐related proteins and induced significant changes in lipid peroxidation in CAL27‐RR cells. Fig. S11. Statins enhance the efficacy of radiation therapy in a xenograft mouse model of CAL27‐RR. [file MOL2-19-540-s001.zip › mol213720-sup-0002-Supplementary_Figure_1.TIF]

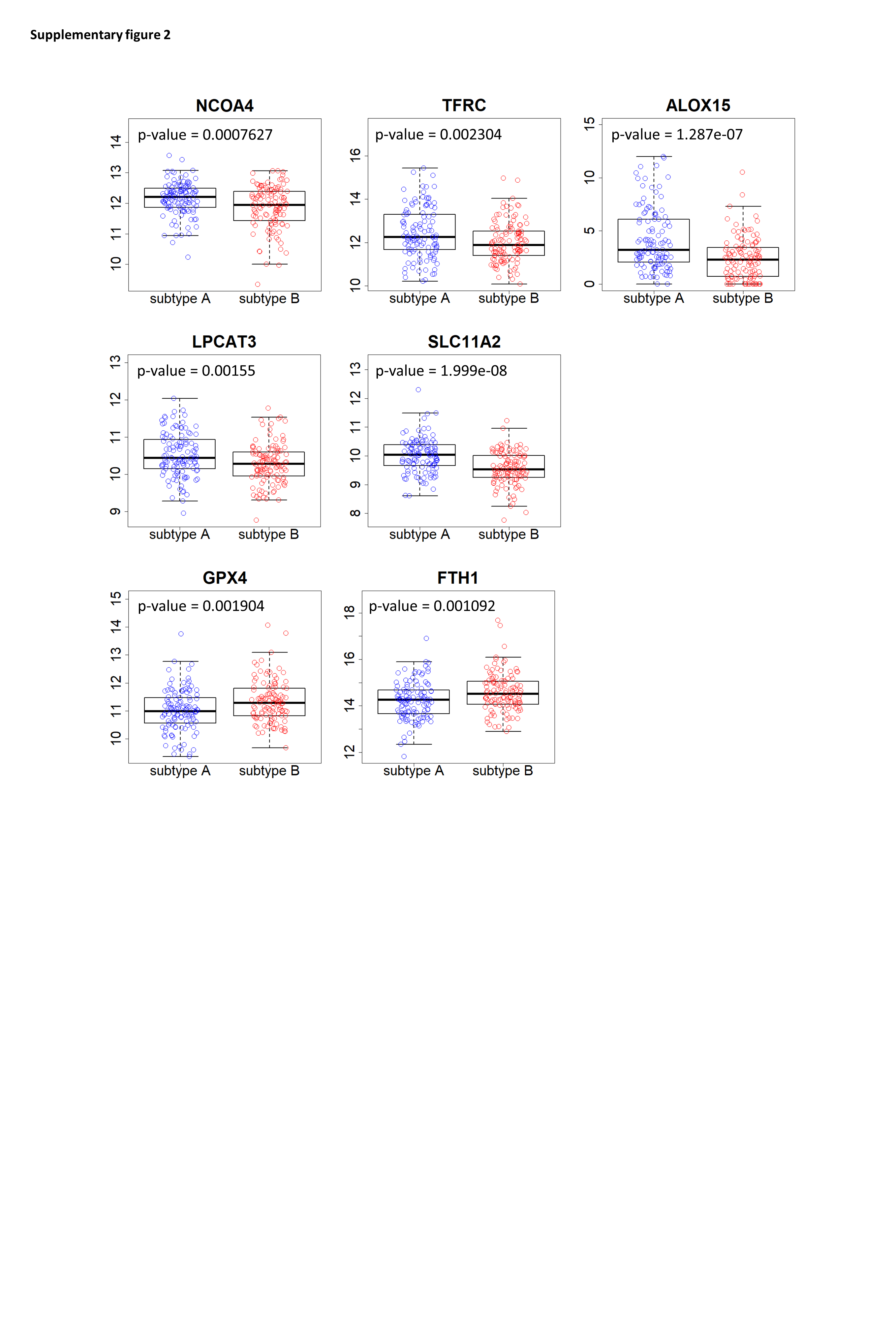

Supplement: Supplementary file 1 — Fig. S1. Ferroptosis‐related gene signature (FRGS) does not show predictive power for prognosis in HPV‐positive HNSCC cohort. Fig. S2. The expression of ferroptosis‐related genes is elevated in subtype A than subtype B. Fig. S3. Validation of the ferroptosis‐related gene signature was conducted in additional cohorts to ensure its robustness and reliability. Fig. S4. Sensitivity of HNSCC cell lines to radiation treatment. Fig. S5. Statins exert a regulatory effect on the sensitivity of cells to radiation and the expression of proteins involved in ferroptosis. Fig. S6. Ferroptosis is related with radioresistance in HNSCC cells. Fig. S7. Lipid peroxidation changes upon radiation, statin, or Fer‐1 treatments. Fig. S8. The application of Fer‐1 counteracts the radiosensitizing effects of statins in subtype B cells, specifically in SNU1076 and YD38 cells. Fig. S9. CAL27‐RR cells showed inhibited ferroptosis than CAL27‐P. Fig. S10. Statins modulated the protein levels of ferroptosis‐related proteins and induced significant changes in lipid peroxidation in CAL27‐RR cells. Fig. S11. Statins enhance the efficacy of radiation therapy in a xenograft mouse model of CAL27‐RR. [file MOL2-19-540-s001.zip › mol213720-sup-0003-Supplementary_Figure_2.TIF]

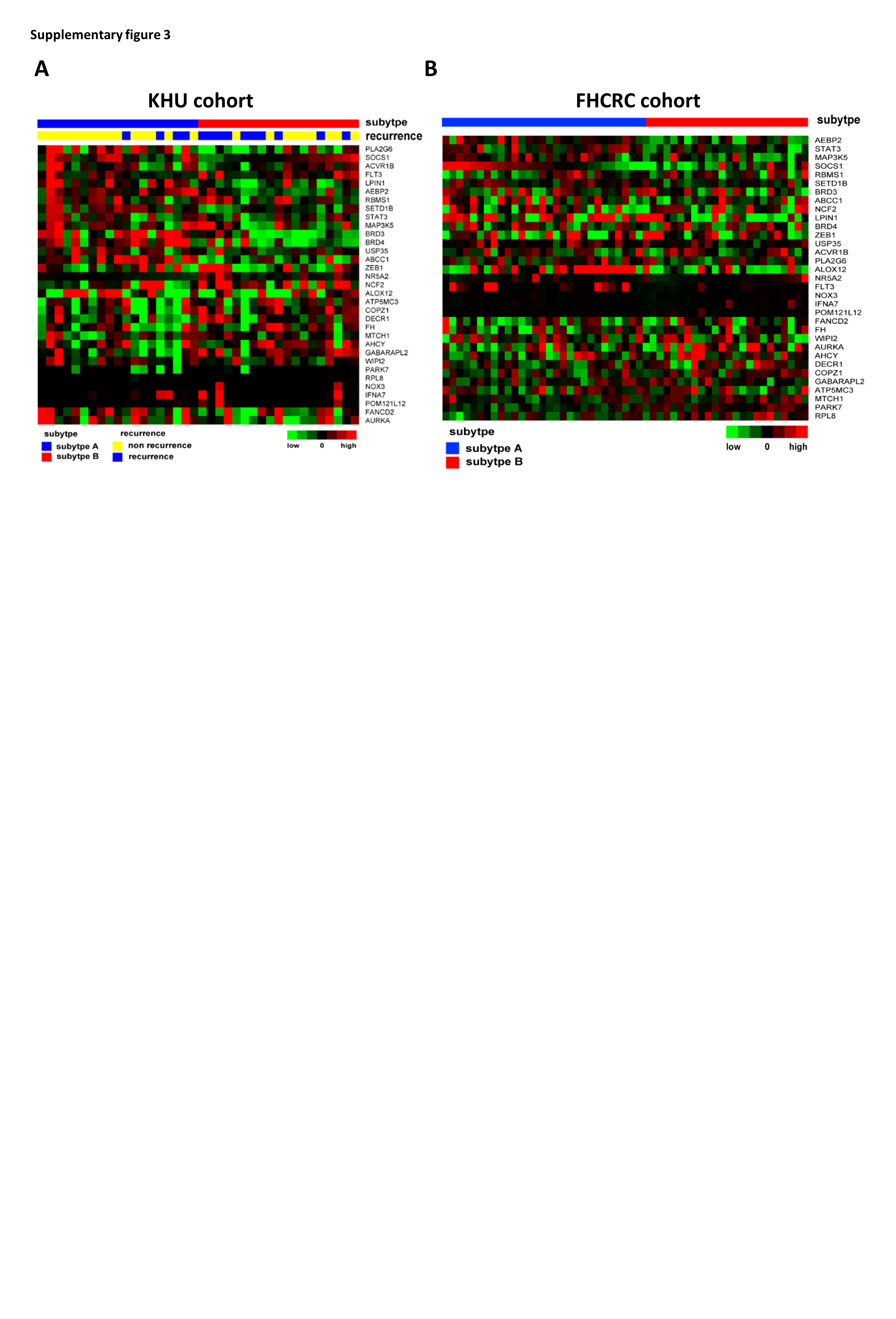

Supplement: Supplementary file 1 — Fig. S1. Ferroptosis‐related gene signature (FRGS) does not show predictive power for prognosis in HPV‐positive HNSCC cohort. Fig. S2. The expression of ferroptosis‐related genes is elevated in subtype A than subtype B. Fig. S3. Validation of the ferroptosis‐related gene signature was conducted in additional cohorts to ensure its robustness and reliability. Fig. S4. Sensitivity of HNSCC cell lines to radiation treatment. Fig. S5. Statins exert a regulatory effect on the sensitivity of cells to radiation and the expression of proteins involved in ferroptosis. Fig. S6. Ferroptosis is related with radioresistance in HNSCC cells. Fig. S7. Lipid peroxidation changes upon radiation, statin, or Fer‐1 treatments. Fig. S8. The application of Fer‐1 counteracts the radiosensitizing effects of statins in subtype B cells, specifically in SNU1076 and YD38 cells. Fig. S9. CAL27‐RR cells showed inhibited ferroptosis than CAL27‐P. Fig. S10. Statins modulated the protein levels of ferroptosis‐related proteins and induced significant changes in lipid peroxidation in CAL27‐RR cells. Fig. S11. Statins enhance the efficacy of radiation therapy in a xenograft mouse model of CAL27‐RR. [file MOL2-19-540-s001.zip › mol213720-sup-0004-Supplementary_Figure_3.TIF]

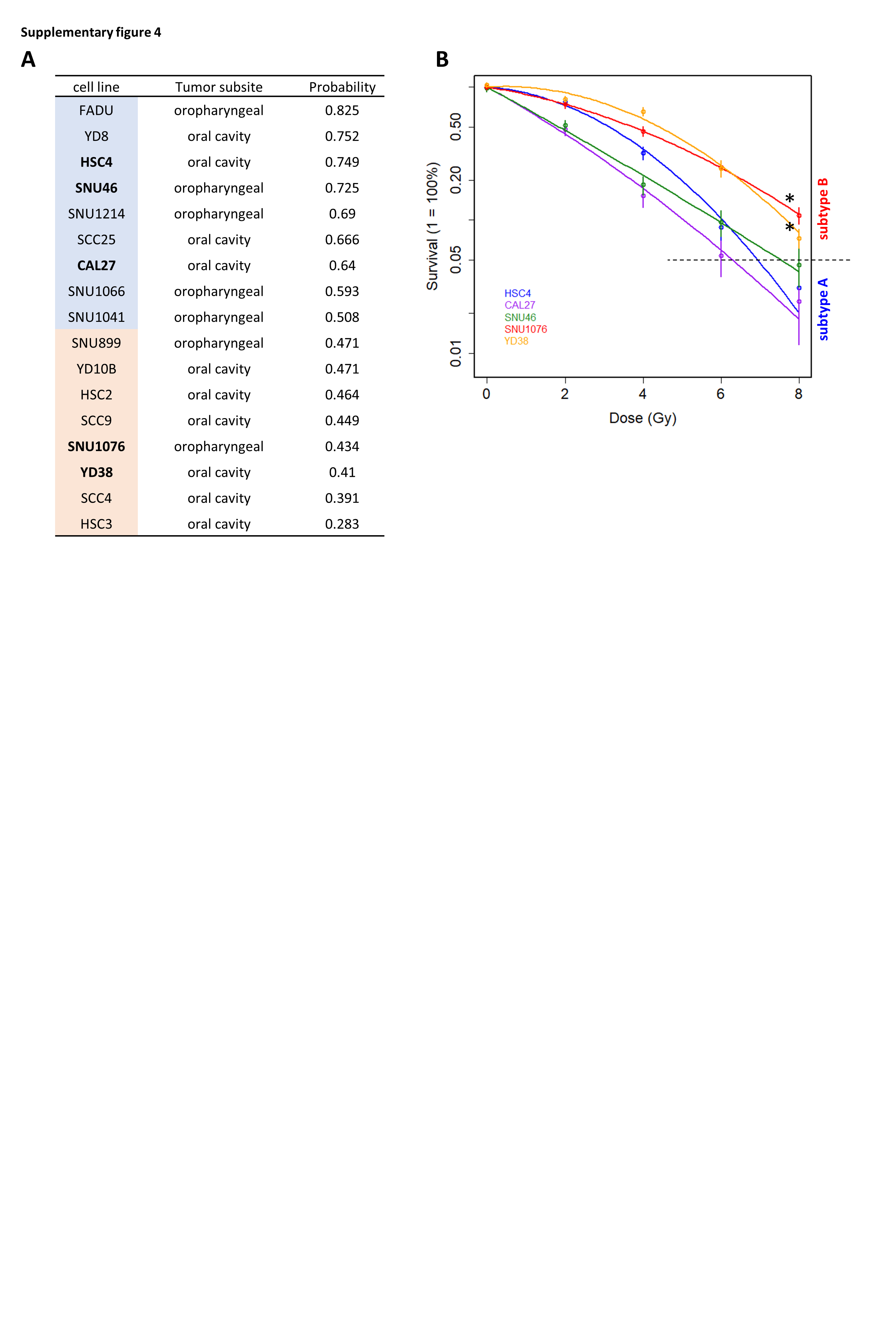

Supplement: Supplementary file 1 — Fig. S1. Ferroptosis‐related gene signature (FRGS) does not show predictive power for prognosis in HPV‐positive HNSCC cohort. Fig. S2. The expression of ferroptosis‐related genes is elevated in subtype A than subtype B. Fig. S3. Validation of the ferroptosis‐related gene signature was conducted in additional cohorts to ensure its robustness and reliability. Fig. S4. Sensitivity of HNSCC cell lines to radiation treatment. Fig. S5. Statins exert a regulatory effect on the sensitivity of cells to radiation and the expression of proteins involved in ferroptosis. Fig. S6. Ferroptosis is related with radioresistance in HNSCC cells. Fig. S7. Lipid peroxidation changes upon radiation, statin, or Fer‐1 treatments. Fig. S8. The application of Fer‐1 counteracts the radiosensitizing effects of statins in subtype B cells, specifically in SNU1076 and YD38 cells. Fig. S9. CAL27‐RR cells showed inhibited ferroptosis than CAL27‐P. Fig. S10. Statins modulated the protein levels of ferroptosis‐related proteins and induced significant changes in lipid peroxidation in CAL27‐RR cells. Fig. S11. Statins enhance the efficacy of radiation therapy in a xenograft mouse model of CAL27‐RR. [file MOL2-19-540-s001.zip › mol213720-sup-0005-Supplementary_Figure_4.TIF]

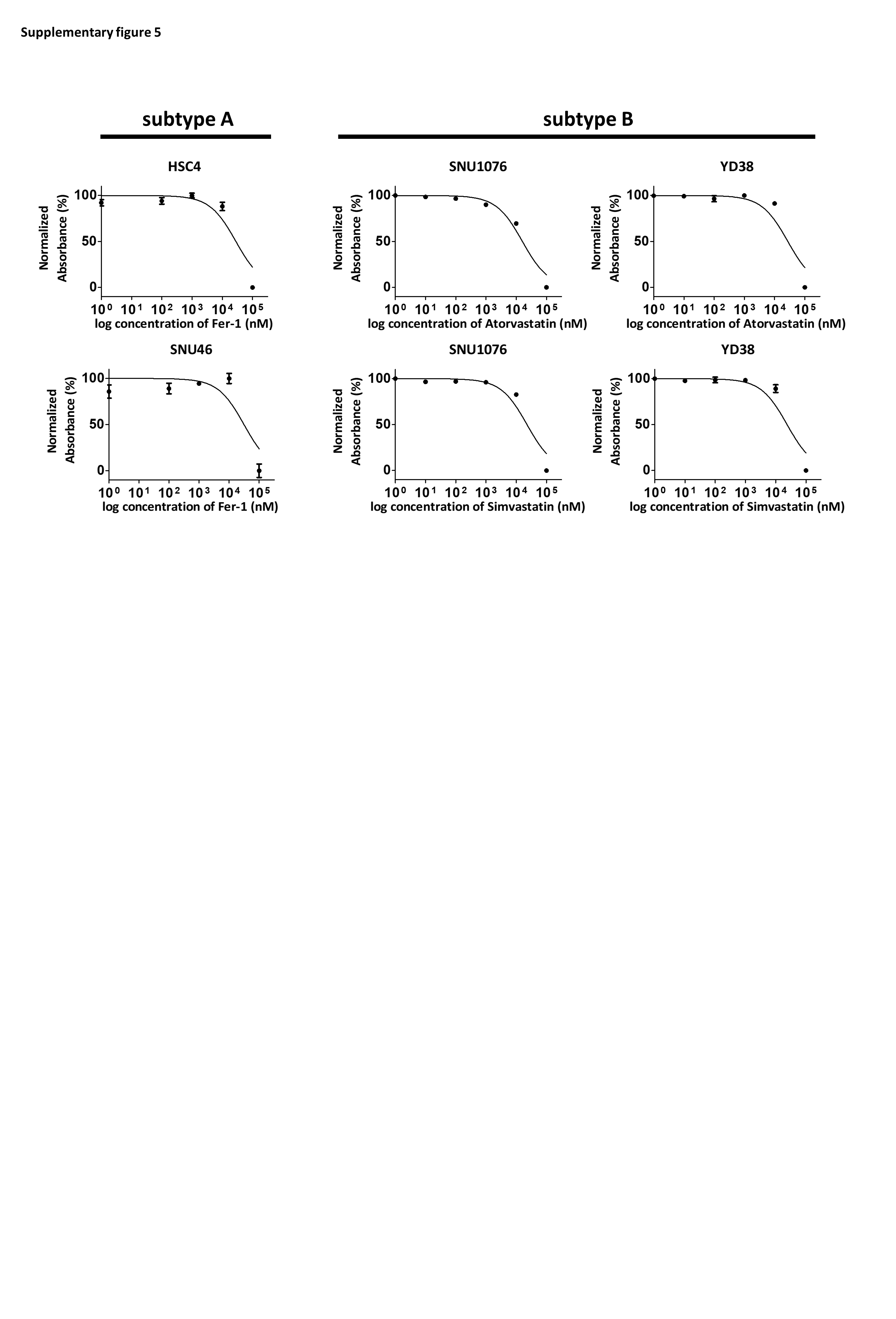

Supplement: Supplementary file 1 — Fig. S1. Ferroptosis‐related gene signature (FRGS) does not show predictive power for prognosis in HPV‐positive HNSCC cohort. Fig. S2. The expression of ferroptosis‐related genes is elevated in subtype A than subtype B. Fig. S3. Validation of the ferroptosis‐related gene signature was conducted in additional cohorts to ensure its robustness and reliability. Fig. S4. Sensitivity of HNSCC cell lines to radiation treatment. Fig. S5. Statins exert a regulatory effect on the sensitivity of cells to radiation and the expression of proteins involved in ferroptosis. Fig. S6. Ferroptosis is related with radioresistance in HNSCC cells. Fig. S7. Lipid peroxidation changes upon radiation, statin, or Fer‐1 treatments. Fig. S8. The application of Fer‐1 counteracts the radiosensitizing effects of statins in subtype B cells, specifically in SNU1076 and YD38 cells. Fig. S9. CAL27‐RR cells showed inhibited ferroptosis than CAL27‐P. Fig. S10. Statins modulated the protein levels of ferroptosis‐related proteins and induced significant changes in lipid peroxidation in CAL27‐RR cells. Fig. S11. Statins enhance the efficacy of radiation therapy in a xenograft mouse model of CAL27‐RR. [file MOL2-19-540-s001.zip › mol213720-sup-0006-Supplementary_Figure_5.TIF]

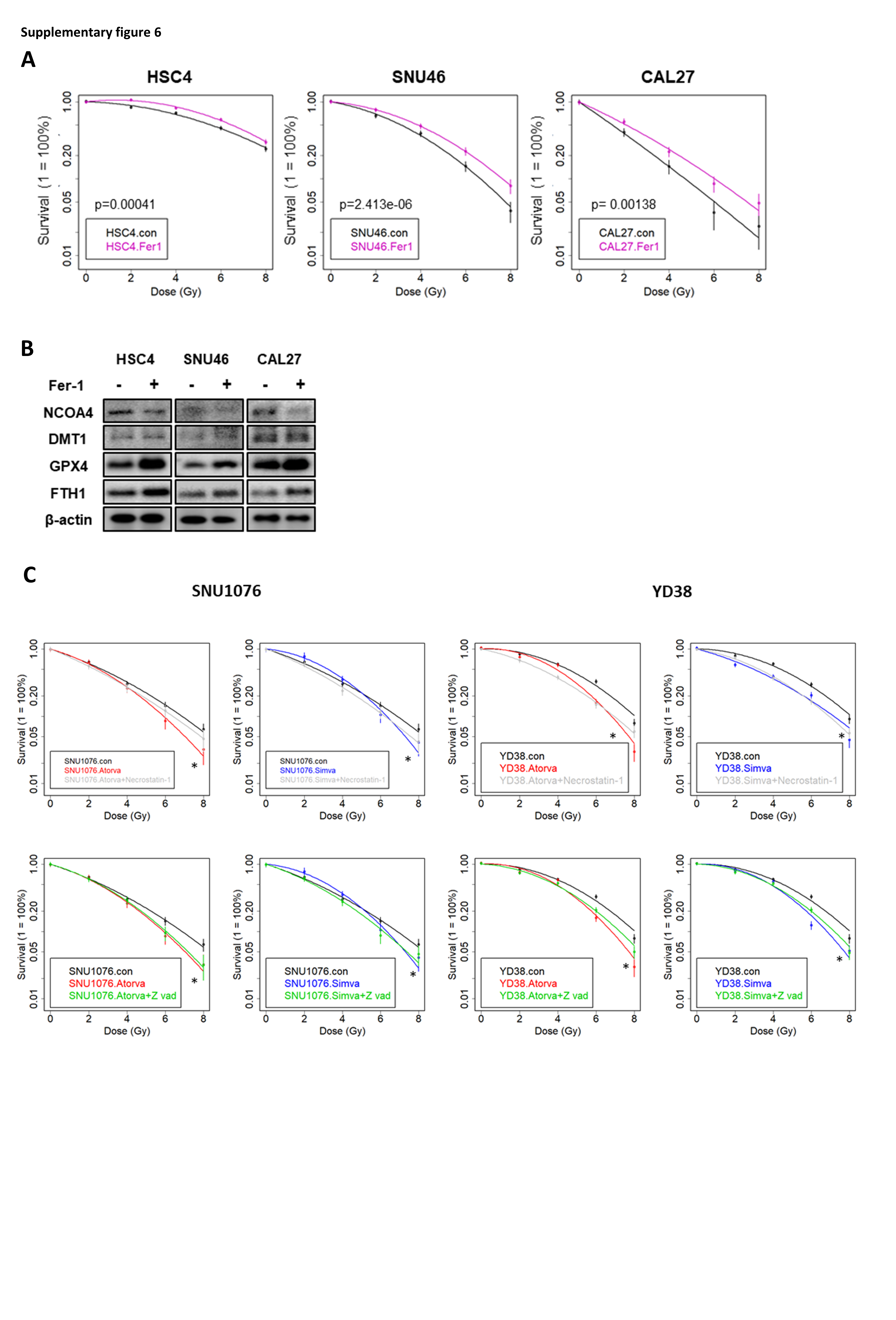

Supplement: Supplementary file 1 — Fig. S1. Ferroptosis‐related gene signature (FRGS) does not show predictive power for prognosis in HPV‐positive HNSCC cohort. Fig. S2. The expression of ferroptosis‐related genes is elevated in subtype A than subtype B. Fig. S3. Validation of the ferroptosis‐related gene signature was conducted in additional cohorts to ensure its robustness and reliability. Fig. S4. Sensitivity of HNSCC cell lines to radiation treatment. Fig. S5. Statins exert a regulatory effect on the sensitivity of cells to radiation and the expression of proteins involved in ferroptosis. Fig. S6. Ferroptosis is related with radioresistance in HNSCC cells. Fig. S7. Lipid peroxidation changes upon radiation, statin, or Fer‐1 treatments. Fig. S8. The application of Fer‐1 counteracts the radiosensitizing effects of statins in subtype B cells, specifically in SNU1076 and YD38 cells. Fig. S9. CAL27‐RR cells showed inhibited ferroptosis than CAL27‐P. Fig. S10. Statins modulated the protein levels of ferroptosis‐related proteins and induced significant changes in lipid peroxidation in CAL27‐RR cells. Fig. S11. Statins enhance the efficacy of radiation therapy in a xenograft mouse model of CAL27‐RR. [file MOL2-19-540-s001.zip › mol213720-sup-0007-Supplementary_Figure_6.TIF]

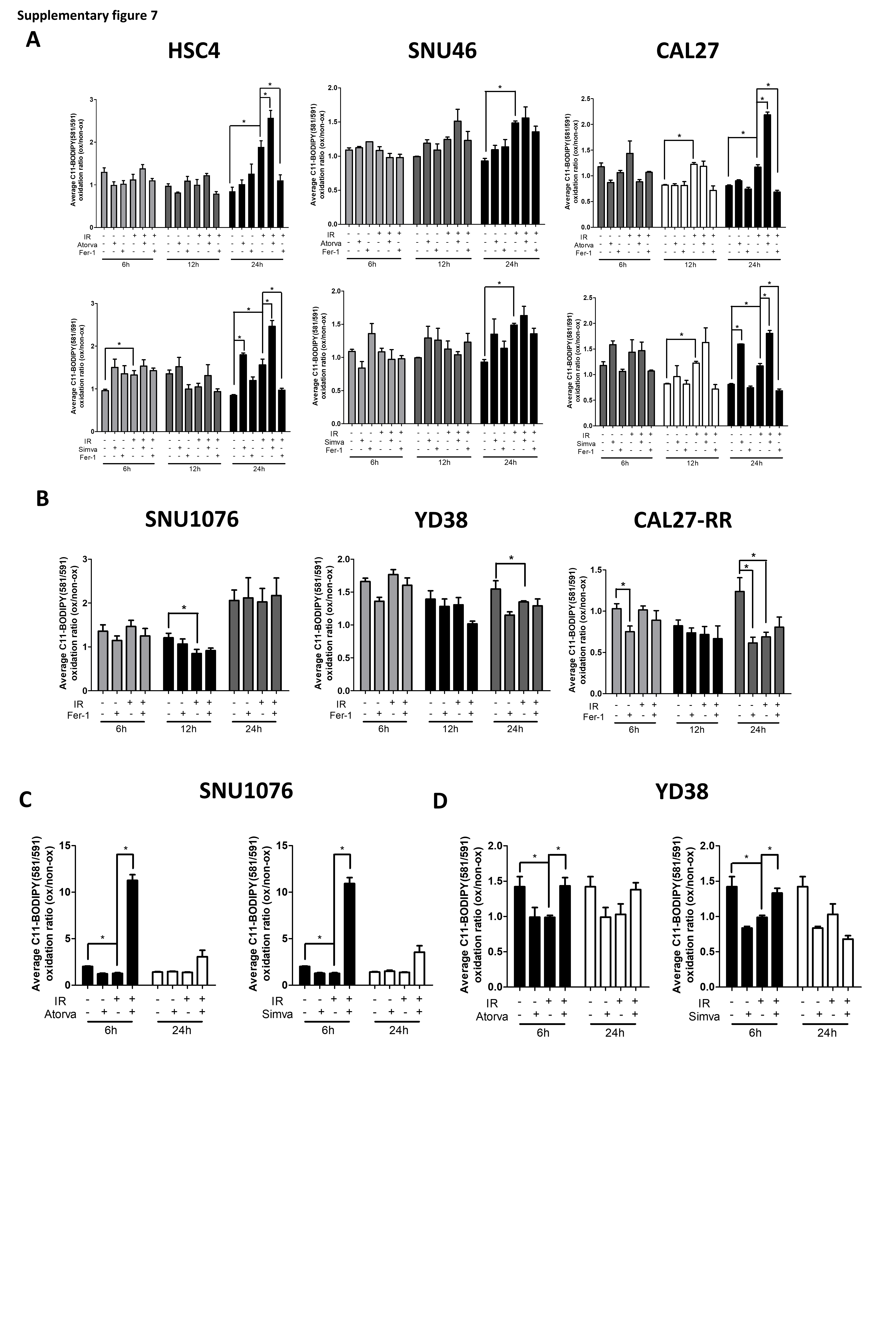

Supplement: Supplementary file 1 — Fig. S1. Ferroptosis‐related gene signature (FRGS) does not show predictive power for prognosis in HPV‐positive HNSCC cohort. Fig. S2. The expression of ferroptosis‐related genes is elevated in subtype A than subtype B. Fig. S3. Validation of the ferroptosis‐related gene signature was conducted in additional cohorts to ensure its robustness and reliability. Fig. S4. Sensitivity of HNSCC cell lines to radiation treatment. Fig. S5. Statins exert a regulatory effect on the sensitivity of cells to radiation and the expression of proteins involved in ferroptosis. Fig. S6. Ferroptosis is related with radioresistance in HNSCC cells. Fig. S7. Lipid peroxidation changes upon radiation, statin, or Fer‐1 treatments. Fig. S8. The application of Fer‐1 counteracts the radiosensitizing effects of statins in subtype B cells, specifically in SNU1076 and YD38 cells. Fig. S9. CAL27‐RR cells showed inhibited ferroptosis than CAL27‐P. Fig. S10. Statins modulated the protein levels of ferroptosis‐related proteins and induced significant changes in lipid peroxidation in CAL27‐RR cells. Fig. S11. Statins enhance the efficacy of radiation therapy in a xenograft mouse model of CAL27‐RR. [file MOL2-19-540-s001.zip › mol213720-sup-0008-Supplementary_Figure_7.TIF]

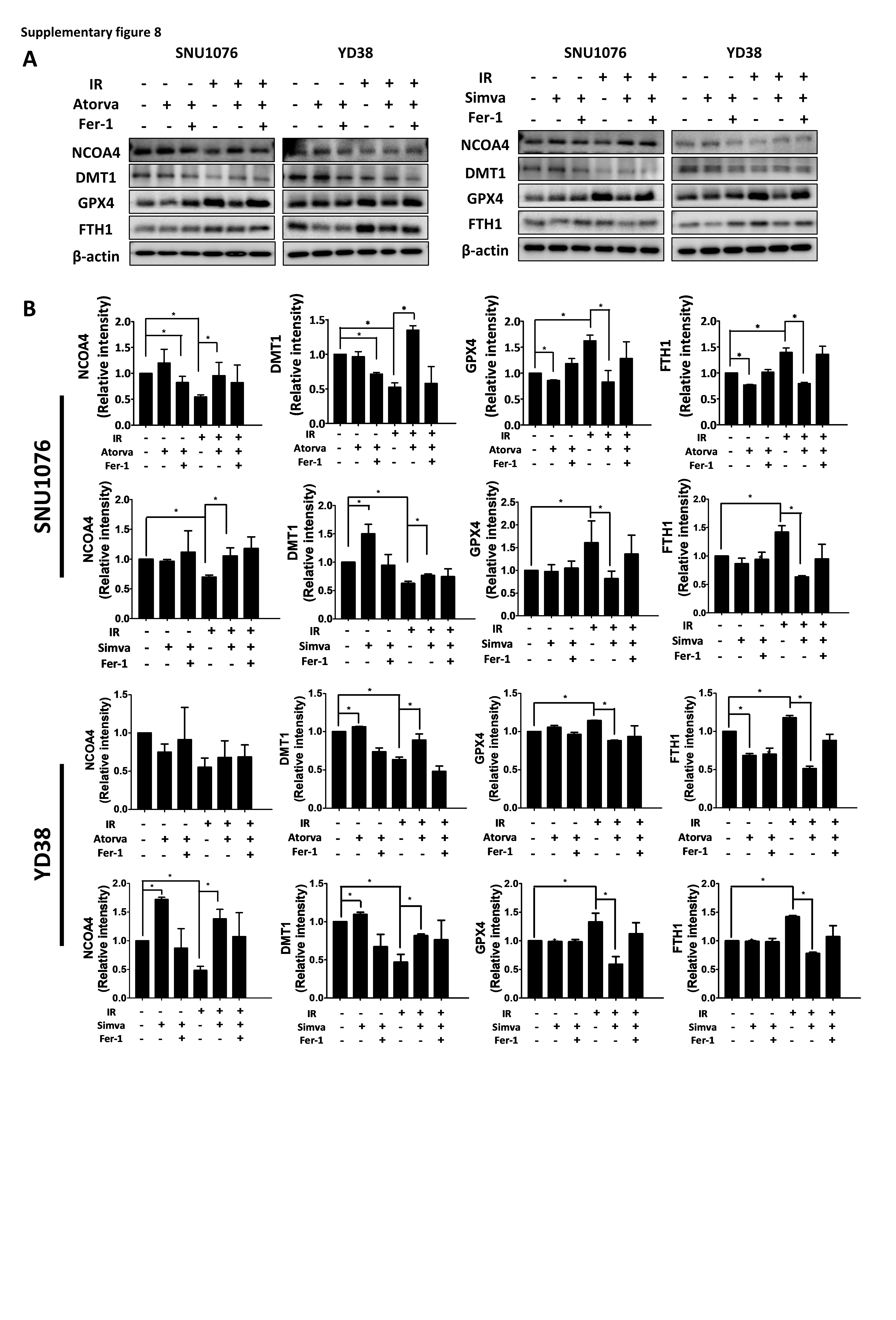

Supplement: Supplementary file 1 — Fig. S1. Ferroptosis‐related gene signature (FRGS) does not show predictive power for prognosis in HPV‐positive HNSCC cohort. Fig. S2. The expression of ferroptosis‐related genes is elevated in subtype A than subtype B. Fig. S3. Validation of the ferroptosis‐related gene signature was conducted in additional cohorts to ensure its robustness and reliability. Fig. S4. Sensitivity of HNSCC cell lines to radiation treatment. Fig. S5. Statins exert a regulatory effect on the sensitivity of cells to radiation and the expression of proteins involved in ferroptosis. Fig. S6. Ferroptosis is related with radioresistance in HNSCC cells. Fig. S7. Lipid peroxidation changes upon radiation, statin, or Fer‐1 treatments. Fig. S8. The application of Fer‐1 counteracts the radiosensitizing effects of statins in subtype B cells, specifically in SNU1076 and YD38 cells. Fig. S9. CAL27‐RR cells showed inhibited ferroptosis than CAL27‐P. Fig. S10. Statins modulated the protein levels of ferroptosis‐related proteins and induced significant changes in lipid peroxidation in CAL27‐RR cells. Fig. S11. Statins enhance the efficacy of radiation therapy in a xenograft mouse model of CAL27‐RR. [file MOL2-19-540-s001.zip › mol213720-sup-0009-Supplementary_Figure_8.TIF]

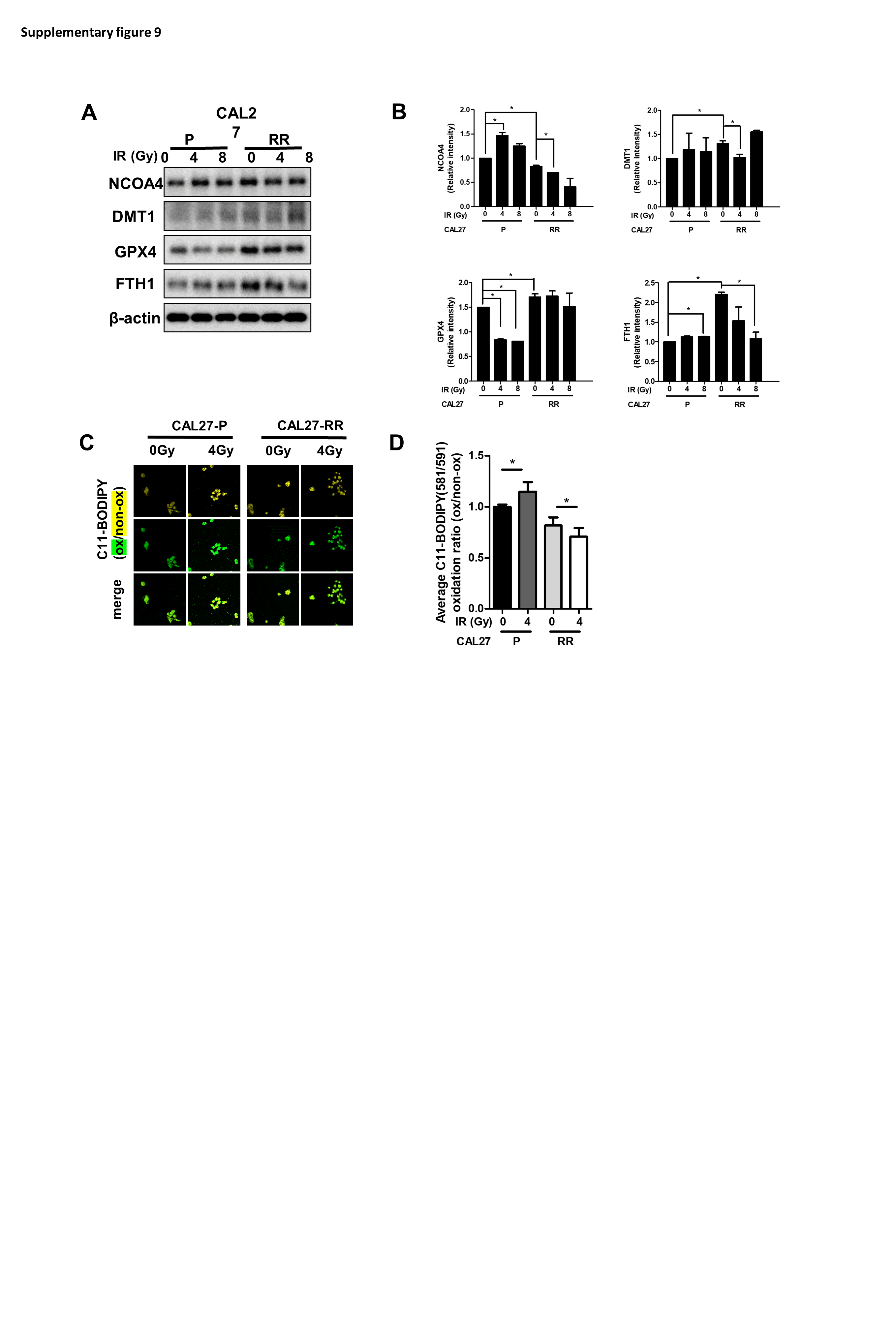

Supplement: Supplementary file 1 — Fig. S1. Ferroptosis‐related gene signature (FRGS) does not show predictive power for prognosis in HPV‐positive HNSCC cohort. Fig. S2. The expression of ferroptosis‐related genes is elevated in subtype A than subtype B. Fig. S3. Validation of the ferroptosis‐related gene signature was conducted in additional cohorts to ensure its robustness and reliability. Fig. S4. Sensitivity of HNSCC cell lines to radiation treatment. Fig. S5. Statins exert a regulatory effect on the sensitivity of cells to radiation and the expression of proteins involved in ferroptosis. Fig. S6. Ferroptosis is related with radioresistance in HNSCC cells. Fig. S7. Lipid peroxidation changes upon radiation, statin, or Fer‐1 treatments. Fig. S8. The application of Fer‐1 counteracts the radiosensitizing effects of statins in subtype B cells, specifically in SNU1076 and YD38 cells. Fig. S9. CAL27‐RR cells showed inhibited ferroptosis than CAL27‐P. Fig. S10. Statins modulated the protein levels of ferroptosis‐related proteins and induced significant changes in lipid peroxidation in CAL27‐RR cells. Fig. S11. Statins enhance the efficacy of radiation therapy in a xenograft mouse model of CAL27‐RR. [file MOL2-19-540-s001.zip › mol213720-sup-0010-Supplementary_Figure_9.TIF]

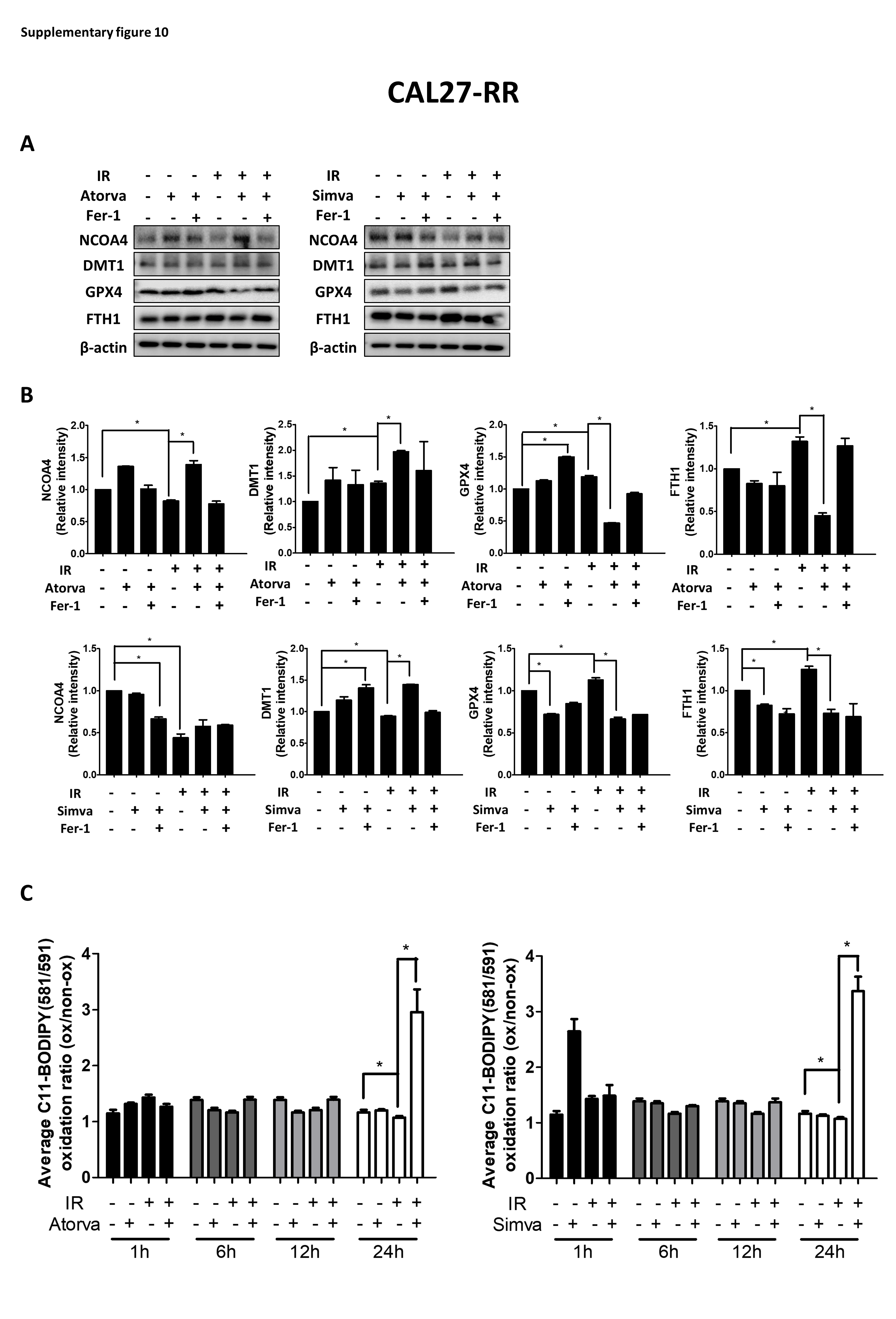

Supplement: Supplementary file 1 — Fig. S1. Ferroptosis‐related gene signature (FRGS) does not show predictive power for prognosis in HPV‐positive HNSCC cohort. Fig. S2. The expression of ferroptosis‐related genes is elevated in subtype A than subtype B. Fig. S3. Validation of the ferroptosis‐related gene signature was conducted in additional cohorts to ensure its robustness and reliability. Fig. S4. Sensitivity of HNSCC cell lines to radiation treatment. Fig. S5. Statins exert a regulatory effect on the sensitivity of cells to radiation and the expression of proteins involved in ferroptosis. Fig. S6. Ferroptosis is related with radioresistance in HNSCC cells. Fig. S7. Lipid peroxidation changes upon radiation, statin, or Fer‐1 treatments. Fig. S8. The application of Fer‐1 counteracts the radiosensitizing effects of statins in subtype B cells, specifically in SNU1076 and YD38 cells. Fig. S9. CAL27‐RR cells showed inhibited ferroptosis than CAL27‐P. Fig. S10. Statins modulated the protein levels of ferroptosis‐related proteins and induced significant changes in lipid peroxidation in CAL27‐RR cells. Fig. S11. Statins enhance the efficacy of radiation therapy in a xenograft mouse model of CAL27‐RR. [file MOL2-19-540-s001.zip › mol213720-sup-0011-Supplementary_Figure_10.TIF]

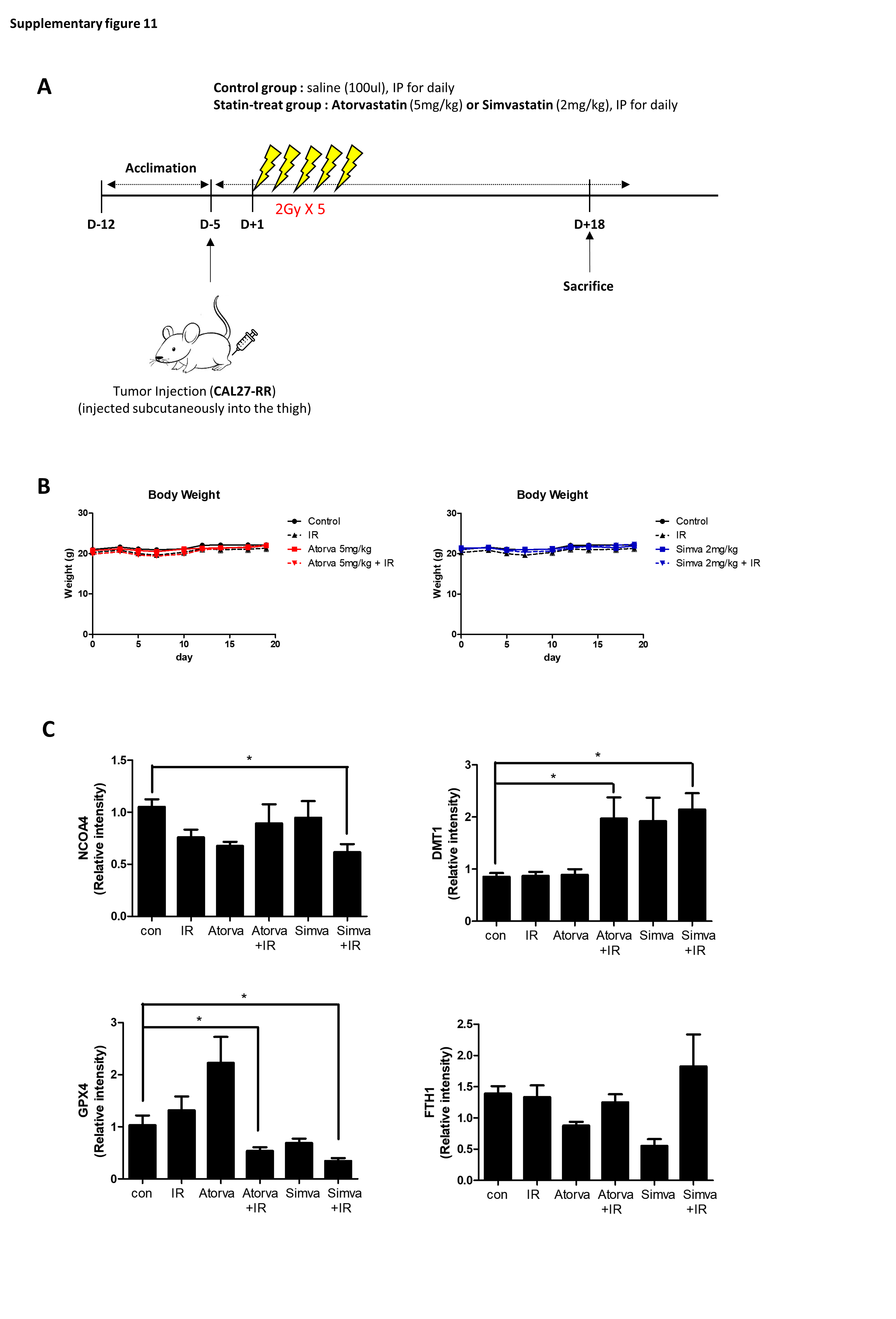

Supplement: Supplementary file 1 — Fig. S1. Ferroptosis‐related gene signature (FRGS) does not show predictive power for prognosis in HPV‐positive HNSCC cohort. Fig. S2. The expression of ferroptosis‐related genes is elevated in subtype A than subtype B. Fig. S3. Validation of the ferroptosis‐related gene signature was conducted in additional cohorts to ensure its robustness and reliability. Fig. S4. Sensitivity of HNSCC cell lines to radiation treatment. Fig. S5. Statins exert a regulatory effect on the sensitivity of cells to radiation and the expression of proteins involved in ferroptosis. Fig. S6. Ferroptosis is related with radioresistance in HNSCC cells. Fig. S7. Lipid peroxidation changes upon radiation, statin, or Fer‐1 treatments. Fig. S8. The application of Fer‐1 counteracts the radiosensitizing effects of statins in subtype B cells, specifically in SNU1076 and YD38 cells. Fig. S9. CAL27‐RR cells showed inhibited ferroptosis than CAL27‐P. Fig. S10. Statins modulated the protein levels of ferroptosis‐related proteins and induced significant changes in lipid peroxidation in CAL27‐RR cells. Fig. S11. Statins enhance the efficacy of radiation therapy in a xenograft mouse model of CAL27‐RR. [file MOL2-19-540-s001.zip › mol213720-sup-0012-Supplementary_Figure_11.TIF]
