# Supplementary material for: Targeting ferroptosis for improved radiotherapy outcomes in HPV‐negative head and neck squamous cell carcinoma
Source: Mol Oncol. 2024 Sep 19;19(2):540–57. doi: 10.1002/1878-0261.13720 (PMC11792990; doi:10.1002/1878-0261.13720)
Supplement: Supplementary file 2 — Table S1. Head and neck squamous cell carcinoma patient's information of TCGA, KHU, and FHCRC cohort. All patients have HPV‐negative cancer and received RT. [file MOL2-19-540-s004.docx]

**Supplementary table 1.** Head and neck squamous cell carcinoma patient’s information of TCGA, KHU, and FHCRC cohort. All patients have HPV-negative cancer and received RT.

|  | **TCGA** | **KHU** | **FHCRC** |
| --- | --- | --- | --- |
| **Number of patients** | 228 | 39 | 53 |
| **Gender** |  |  |  |
| Male | 176 | 34 | 35 |
| Female | 52 | 5 | 18 |
| **Age** |  |  |  |
| ≥60 years old | 122 | 26 | 22 |
| <60 years old | 106 | 11 | 31 |
| **Primary tumor** |  |  |  |
| T1 | 15 | 3 |  |
| T2 | 51 | 8 |  |
| T3 | 59 | 5 |  |
| T4 | 103 | 22 |  |
| **Regional lymph node** |  |  |  |
| N0 | 89 | 16 |  |
| N1 | 36 | 8 |  |
| N2 | 101 | 14 |  |
| N3 | 2 | 0 |  |
| **Stage** |  |  |  |
| I | 6 | 1 | 8 |
| II | 15 | 4 |  |
| III | 39 | 4 | 45 |
| IV | 168 | 18 |  |
| **Smoking** |  |  |  |
| Yes | 171 | 29 |  |
| No | 53 | 9 |  |
| **Alcohol** |  |  |  |
| Yes | 165 |  |  |
| No | 61 |  |  |
