# Supplementary material for: Targeting ferroptosis for improved radiotherapy outcomes in HPV‐negative head and neck squamous cell carcinoma
Source: Mol Oncol. 2024 Sep 19;19(2):540–57. doi: 10.1002/1878-0261.13720 (PMC11792990; doi:10.1002/1878-0261.13720)
Supplement: Supplementary file 3 — Table S2. The list of 33 ferroptosis‐related genes comprising FRGS. [file MOL2-19-540-s002.docx]

**Supplementary table 2.** The list of 33 Ferroptosis-related genes comprising FRGS.

| **Gene name** | **Z score** | **P value** | **Cox value** | **coefficient** | **CI Lower** | **CI Upper** | **FDR** |
| --- | --- | --- | --- | --- | --- | --- | --- |
| NOX3 | 3.505 | 0 | 12.796 | 2.549 | 3.077 | 53.225 | 0.226675 |
| SOCS1 | -3.257 | 0.001 | 0.64 | -0.447 | 0.489 | 0.837 | 0.227759 |
| IFNA7 | 3.189 | 0.001 | 5.21 | 1.65 | 1.889 | 14.364 | 0.227759 |
| MTCH1 | 3.116 | 0.002 | 2.733 | 1.005 | 1.452 | 5.143 | 0.227759 |
| NR5A2 | -2.846 | 0.004 | 0.659 | -0.417 | 0.495 | 0.878 | 0.439639 |
| POM121L12 | 2.697 | 0.007 | 4.721 | 1.552 | 1.528 | 14.582 | 0.579352 |
| ABCC1 | -2.645 | 0.008 | 0.713 | -0.338 | 0.555 | 0.916 | 0.580677 |
| FH | 2.584 | 0.01 | 2 | 0.693 | 1.182 | 3.383 | 0.6062 |
| PARK7 | 2.464 | 0.014 | 1.676 | 0.516 | 1.111 | 2.527 | 0.646701 |
| BRD4 | -2.426 | 0.015 | 0.637 | -0.451 | 0.443 | 0.917 | 0.646701 |
| ATP5MC3 | 2.383 | 0.017 | 1.514 | 0.415 | 1.076 | 2.129 | 0.646701 |
| SETD1B | -2.37 | 0.018 | 0.608 | -0.497 | 0.403 | 0.918 | 0.646701 |
| FLT3 | -2.338 | 0.019 | 0.83 | -0.186 | 0.711 | 0.97 | 0.646701 |
| STAT3 | -2.334 | 0.02 | 0.587 | -0.532 | 0.375 | 0.918 | 0.646701 |
| PLA2G6 | -2.313 | 0.021 | 0.725 | -0.322 | 0.552 | 0.952 | 0.646701 |
| NCF2 | -2.289 | 0.022 | 0.744 | -0.296 | 0.577 | 0.958 | 0.646701 |
| COPZ1 | 2.288 | 0.022 | 1.987 | 0.687 | 1.104 | 3.578 | 0.646701 |
| BRD3 | -2.266 | 0.023 | 0.679 | -0.387 | 0.486 | 0.949 | 0.648144 |
| ACVR1B | -2.194 | 0.028 | 0.668 | -0.404 | 0.466 | 0.958 | 0.711967 |
| WIPI2 | 2.183 | 0.029 | 1.828 | 0.603 | 1.064 | 3.143 | 0.711967 |
| DECR1 | 2.169 | 0.03 | 1.524 | 0.422 | 1.041 | 2.231 | 0.711967 |
| AHCY | 2.108 | 0.035 | 1.589 | 0.463 | 1.033 | 2.444 | 0.72679 |
| GABARAPL2 | 2.11 | 0.035 | 1.654 | 0.503 | 1.036 | 2.64 | 0.72679 |
| ALOX12 | -2.062 | 0.039 | 0.895 | -0.111 | 0.806 | 0.995 | 0.72679 |
| AEBP2 | -2.069 | 0.039 | 0.702 | -0.354 | 0.502 | 0.982 | 0.72679 |
| LPIN1 | -2.047 | 0.041 | 0.761 | -0.273 | 0.586 | 0.988 | 0.72679 |
| ZEB1 | -2.028 | 0.043 | 0.812 | -0.208 | 0.665 | 0.993 | 0.72679 |
| FANCD2 | 2.017 | 0.044 | 1.394 | 0.332 | 1.009 | 1.924 | 0.72679 |
| AURKA | 2.019 | 0.044 | 1.385 | 0.326 | 1.01 | 1.901 | 0.72679 |
| RBMS1 | -1.991 | 0.046 | 0.629 | -0.464 | 0.398 | 0.993 | 0.72679 |
| RPL8 | 1.983 | 0.047 | 1.322 | 0.279 | 1.003 | 1.742 | 0.72679 |
| USP35 | -1.975 | 0.048 | 0.719 | -0.33 | 0.518 | 0.997 | 0.72679 |
| MAP3K5 | -1.976 | 0.048 | 0.726 | -0.321 | 0.528 | 0.997 | 0.72679 |

CI, Confidence interval; FDR, False discovery rate
