# Supplementary material for: Targeting ferroptosis for improved radiotherapy outcomes in HPV‐negative head and neck squamous cell carcinoma
Source: Mol Oncol. 2024 Sep 19;19(2):540–57. doi: 10.1002/1878-0261.13720 (PMC11792990; doi:10.1002/1878-0261.13720)
Supplement: Supplementary file 4 — Table S3. Patients' information between subtype A and subtype B were listed in cohorts from TCGA, KHU, and FHCRC. [file MOL2-19-540-s003.docx]

**Supplementary table 3.** Patients' information between subtype A and subtype B were listed in cohorts from TCGA, KHU, and FHCRC.

|  | **TCGA** | |  | **KHU** | |  | **FHCRC** | |  |
| --- | --- | --- | --- | --- | --- | --- | --- | --- | --- |
|  | **Subtype A** | **Subtype B** | **p-value** | **Subtype A** | **Subtype B** | **p-value** | **Subtype A** | **Subtype B** | **p-value** |
| **Number of patients** | 114 | 114 |  | 20 | 19 |  | 30 | 23 |  |
| **Gender** |  |  | 0.87 |  |  | 1 |  |  | **0.03** |
| Male | 89 | 87 |  | 17 | 17 |  | 24 | 11 |  |
| Female | 25 | 27 |  | 3 | 2 |  | 6 | 12 |  |
| **Age** |  |  | 0.89 |  |  | 0.41 |  |  | 0.56 |
| ≥60 years old | 62 | 60 |  | 15 | 11 |  | 14 | 8 |  |
| <60 years old | 52 | 54 |  | 4 | 7 |  | 16 | 15 |  |
| **Primary tumor** |  |  | 0.05 |  |  | 0.15 |  |  |  |
| T1 | 7 | 0 |  | 0 | 3 |  |  |  |  |
| T2 | 18 | 21 |  | 3 | 5 |  |  |  |  |
| T3 | 31 | 38 |  | 3 | 2 |  |  |  |  |
| T4 | 53 | 52 |  | 14 | 8 |  |  |  |  |
| **Regional lymph node** |  |  | 0.81 |  |  | 0.34 |  |  |  |
| N0 | 47 | 43 |  | 7 | 9 |  |  |  |  |
| N1 | 22 | 26 |  | 6 | 2 |  |  |  |  |
| N2 | 36 | 39 |  | 7 | 7 |  |  |  |  |
| N3 | 2 | 1 |  | 0 | 0 |  |  |  |  |
| **Stage** |  |  | 0.21 |  |  | 0.53 |  |  | 0.43 |
| 0.43I | 4 | 0 |  | 0 | 1 |  | 3 | 5 |  |
| II | 8 | 11 |  | 2 | 2 |  |  |  |  |
| III | 24 | 25 |  | 1 | 3 |  | 27 | 18 |  |
| IV | 73 | 76 |  | 10 | 8 |  |  |  |  |
| **Smoking** |  |  | **0.01** |  |  | 1 |  |  |  |
| Yes | 94 | 77 |  | 15 | 14 |  |  |  |  |
| No | 18 | 35 |  | 5 | 5 |  |  |  |  |
| **Alcohol** |  |  | 0.26 |  |  |  |  |  |  |
| Yes | 79 | 86 |  |  |  |  |  |  |  |
| No | 35 | 26 |  |  |  |  |  |  |  |

Statistical significance was calculated with a chi-square test.

Bold value indicates statistical significance by setting p<0.05.
